# Biophysical and Lipidomic Biomarkers of Cardiac Remodeling Post-Myocardial Infarction in Humans

**DOI:** 10.3390/biom10111471

**Published:** 2020-10-22

**Authors:** Valerie Samouillan, Ignacio Miguel Martinez de Lejarza Samper, Aleyda Benitez Amaro, David Vilades, Jany Dandurand, Josefina Casas, Esther Jorge, David de Gonzalo Calvo, Alberto Gallardo, Enrique Lerma, Jose Maria Guerra, Francesc Carreras, Ruben Leta, Vicenta Llorente Cortes

**Affiliations:** 1 CIRIMAT, Université de Toulouse, Université Paul Sabatier, Equipe PHYPOL, 31062 Toulouse, France; valerie.samouillan@univ-tlse3.fr (V.S.); jany.lods@univ-tlse3.fr (J.D.); 2 Department of Cardiology, Hospital de la Santa Creu i Sant Pau, Biomedical Research Institute Sant Pau (IIB Sant Pau), Universitat Autonoma de Barcelona, 08193 Barcelona, Spain; IMartinezL@santpau.cat (I.M.M.d.L.S.); DVilades@santpau.cat (D.V.); EJorge@santpau.cat (E.J.); JGuerra@santpau.cat (J.M.G.); FCarreras@santpau.cat (F.C.); RLeta@santpau.cat (R.L.); 3 CIBERCV, Institute of Health Carlos III, 28029 Madrid, Spain; 4 Institute of Biomedical Research of Barcelona (IIBB), Spanish National Research Council (CSIC), 08036 Barcelona, Spain; ABenitez@santpau.cat (A.B.A.); david.degonzalo@gmail.com (D.d.G.C.); 5 Group of Lipids and Cardiovascular Pathology, Biomedical Research Institute Sant Pau (IIB Sant Pau), Hospital de la Santa Creu i Sant Pau, 08041 Barcelona, Spain; 6 Research Unit on BioActive Molecules (RUBAM), Department of Biological Chemistry, Institute for Advanced Chemistry of Catalonia (IQAC-CSIC), 08034 Barcelona, Spain; fina.casas@iqac.csic.es; 7 CIBEREHD Institute of Health Carlos III, 28029 Madrid, Spain; 8 Department of Pathology, Hospital de la Santa Creu i Sant Pau, 08041 Barcelona, Spain; AGallardo@santpau.cat (A.G.); ELerma@santpau.cat (E.L.)

**Keywords:** biophysical markers, cardiac remodeling post-MI, lipidomics, Fourier transform infrared spectroscopy, heart failure

## Abstract

Few studies have analyzed the potential of biophysical parameters as markers of cardiac remodeling post-myocardial infarction (MI), particularly in human hearts. Fourier transform infrared spectroscopy (FTIR) illustrates the overall changes in proteins, nucleic acids and lipids in a single signature. The aim of this work was to define the FTIR and lipidomic pattern for human left ventricular remodeling post-MI. A total of nine explanted hearts from ischemic cardiomyopathy patients were collected. Samples from the right ventricle (RV), left ventricle (LV) and infarcted left ventricle (LV INF) were subjected to biophysical (FTIR and differential scanning calorimetry, DSC) and lipidomic (liquid chromatography–high-resolution mass spectrometry, LC–HRMS) studies. FTIR evidenced deep alterations in the myofibers, extracellular matrix proteins, and the hydric response of the LV INF compared to the RV or LV from the same subject. The lipid and esterified lipid FTIR bands were enhanced in LV INF, and both lipid indicators were tightly and positively correlated with remodeling markers such as collagen, lactate, polysaccharides, and glycogen in these samples. Lipidomic analysis revealed an increase in several species of sphingomyelin (SM), hexosylceramide (HexCer), and cholesteryl esters combined with a decrease in glycerophospholipids in the infarcted tissue. Our results validate FTIR indicators and several species of lipids as useful markers of left ventricular remodeling post-MI in humans.

## 1. Introduction

Ischemic heart disease is the primary cause of death in Western countries, and myocardial infarction occupies about 50% of deaths in this group. Despite the important improvements in the management of myocardial infarction, adverse left ventricular remodeling, which occurs in up to 30% of cases following ST-segment elevation myocardial infarction (STEMI), is strongly associated with poor patient outcomes. Chronic left ventricular remodeling (LVR) is, apart from infarct size and infarct wound healing, the primary determinant of heart failure post-myocardial infarction (MI) [1,2]. Adverse LVR after MI involves crucial changes in the composition and organization of the extracellular matrix (ECM) [3]. 

The location, size and shape of MI is commonly determined by imaging techniques at the clinical level or by histopathology at the experimental level. Detailed information about the chemical composition and physical structure of infarct zones post-MI remains limited. Previous studies from our group have shown that Fourier transform infrared (FTIR) spectroscopy has the potential to highlight the main alterations that occur in cardiac remodeling in a post-MI mice model [4], as well as in a pig model of tachycardia-induced dilated cardiomyopathy [5]. The FTIR spectra of freeze-dried mice’s left ventricles showed amides I and II to be the major absorptions bands. Collagen possesses a specific band at 1338 cm^−1^ [6] that can be used to compile a collagen/protein indicator. Finally, the sub-resolution of the FTIR spectra determined by Fourier self-deconvolution (FSD) and the second derivative method in the amide I/II zone is useful for determining the secondary structures of proteins [7]. In a mice model, we showed that an increase in the collagen indicator in the infarcted tissue is associated with the predominance of the triple helical conformation of proteins, evidencing a deep remodeling of this zone. 

In addition to structural remodeling, infarcted tissue undergoes what is called “metabolic remodeling”, which includes a set of metabolic changes that occur in the cardiac tissue exposed to ischemia. These metabolic changes include partial insulin resistance, associated with reduced fatty acid oxidation and impaired mitochondrial biogenesis [8,9], downregulation of metabolic genes [10], and upregulation of lipoprotein receptors that contribute to increasing the intracellular lipids in cardiomyocytes [11,12,13,14]. A high prevalence of myocardial lipids has been found in areas of chronic MI in humans [15]. Patients with cardiac lipid deposition had larger infarctions, as well as decreased wall thickening and impaired endocardial wall motion. The hydrolysis of lipid species such as phospholipids in the membrane of cardiomyocytes during ischemic processes is intimately linked to the pathogenesis of myocardial infarction [16,17]. Clinical studies have identified new circulating metabolites that are derived from phospholipidic metabolism in serum and are useful as potential new biomarkers in cardiac remodeling post-MI [18,19,20,21,22]. To the best of our knowledge, only one lipidomic study has been performed in cardiac infarcted tissues, and was developed using pigs [13]. 

The objective of the current investigation was to identify conformational, biophysical, and lipidomic alterations that are useful as biomarkers of cardiac adverse remodeling in human infarcted hearts.

## 2. Materials and Methods 

### 2.1. Collection of Human Samples

A total of 9 explanted human hearts from ischemic cardiomyopathy patients were collected and immediately processed. These hearts were from patients undergoing cardiac transplantation in the Department of Cardiology. The myocardial samples from the explanted hearts were collected in the Department of Pathology (both departments from Santa Creu I Sant Pau Hospital, Barcelona). Clinical data, electrocardiograms, Doppler echocardiography, hemodynamic studies, and coronary angiography were available for all patients. All patients were functionally classified according to the New York Heart Association (NYHA) criteria and received medical treatment according to the guidelines of the European Society of Cardiology [23] using diuretics 89%, angiotensin-converting enzyme inhibitors 86%, β-blockers 48%, aldosterone antagonists 71%, digoxin 49% and statins 82% (Table 1). 

Hearts were weighed and measured, and samples from the right ventricle (RV) (*n* = 9), left ventricle (LV) (*n* = 9), and infarcted left ventricle (LV INF) (*n* = 9) were excised and frozen at −80 °C for immunohistochemical, biophysical and lipidomic studies. The project was approved by the local Ethics Committee of Hospital de la Santa Creu i Sant Pau, Barcelona, Spain, and was conducted in accordance with the guidelines of the Declaration of Helsinki. All patients gave written informed consent that was obtained according to our institutional guidelines.

### 2.2. Tissue Homogenization and Preparation of the Samples for the Different Studies

One portion of collected tissue was embedded in optimal cutting temperature compound (OCT) and used for immunohistochemical studies. Other portion was frozen under N_2_, pulverized using a mortar and a pestle in liquid nitrogen and used for the biophysical studies. A 5 mg aliquot was freeze dried and used for vibrational characterization and a 25 mg aliquot was used for differential scanning calorimetry (DSC). Another aliquot of pulverized tissue (500 µg) was dissolved in a lysis buffer (Tris-HCl 1 M, KCl 1 M, and protease inhibitors 1 µg/mL) and used for the lipidomic studies.

### 2.3. Immunohistochemical Analysis

Myocardial collagen was immunohistochemically assessed by Sirius Red Staining as previously described [24].

### 2.4. Vibrational Characterization

The Fourier transform infrared spectroscopy/attenuated total reflectance (FTIR/ATR) spectra of the freeze-dried tissues were acquired using a Nicolet 5700 FTIR instrument (Thermo Fisher Scientific, Waltham, MA, USA) equipped with an ATR device with a KBr beam splitter and an MCT/B detector. The ATR accessory used was a Smart Orbit with a type IIA diamond crystal (refractive index 2.4, Thermo Fisher Scientific, Waltham, MA, USA). Samples were directly deposited on the entire active surface of the crystal and gently compressed using a Teflon tip to ensure good contact. For each sample, 80 interferograms were recorded in the 4000–450 cm^−1^ region, co-added and Fourier-transformed to generate an average spectrum of the segmented heart part with a nominal resolution of 1 cm^−1^ using Omnic 8.0 (Thermo Fisher Scientific, Waltham, MA, USA). The single-beam background spectrum was collected from the clean diamond crystal before each experiment, and this background was subtracted from the spectra.

To circumvent the attenuation of penetration depth in the samples at large wave numbers in the ATR mode, spectra were subjected to advanced ATR correction. Then, the spectra were baseline corrected and normalized using the maximum of the amide II peak. These spectra were subsequently used to calculate the integrated band intensities and their ratios. For semi-quantitative comparison between groups, the areas of the different absorption bands were computed from the individual spectrum of each tissue, and the appropriate ratio of areas was used according to the literature data in trans-reflectance or ATR mode [25,26]. Second derivatives were used to enhance the chemical information present in the overlapping infrared absorption bands of the spectra. All spectra processing was performed using Omnic 8.0. The spectra presented for each group were calculated by averaging the spectra of all samples within each group.

### 2.5. Differential Scanning Calorimetry

Calorimetric analyses were performed using a DSC Pyris calorimeter (Perkin Elmer, Waltham, MA). The calorimeter was calibrated using Hg and In resulting in a temperature accuracy of 0.1 °C and an enthalpy accuracy of 0.2 J/g. Fresh samples, 5–10 mg in weight, were set into hermetic aluminum pans and equilibrated at the initial temperature for 5 min before cooling to −100 °C at 10 °C/min. Then, the thermograms were recorded during heating at 10 °C/min until reaching 90 °C. After the DSC measurements were performed, the pans were reweighed to check that they had been correctly sealed.

### 2.6. Lipidomic Analysis of the Heart

Phospholipids (PLs) and sphingolipids (SLs extracts) were prepared and analyzed using the following protocols previously described [27,28], with minor modifications.

#### 2.6.1. Phospholipids and Neutral Lipids 

A 750 µL methanol–chloroform (1:2, *v*/*v*) solution containing standards was transferred to borosilicate glass test tubes with Teflon caps, and 0.25 mL methanol and 0.5 mL chloroform were subsequently added. This mixture was fortified with the internal standards of lipids (200 pmol each). The following standards were added to myocardial samples: 16:0 D31_18:1 phosphocholine, 16:0 D31_18:1 phosphoethanolamine, 16:0 D31-18:1 phosphoserine, 17:0 lyso-phosphocholine, 17:1 lyso-phosphoethanolamine, 17:1 lyso-phosphoserine, 17:0/17:0/17:0 triacylglycerol, and C17:0 cholesteryl ester; 0.2 nmol of each standard (from Avanti Polar Lipids). The samples were vortexed and sonicated until they appeared dispersed and were then incubated at 48 °C overnight. The solvent was removed using a Speed Vac Savant SPD131DDA (Thermo Scientific). Lipids were solubilized in 0.5 mL of methanol and transferred to 1.5 mL Eppendorf tubes and evaporated again. The samples were resuspended in 150 µL of methanol. The tubes were centrifuged at 13,000× *g* for 3 min, and 130 µL of the supernatants was transferred to ultra-performance liquid chromatography (UPLC) vials for injection and analysis. 

#### 2.6.2. Sphingolipids

A 750 µL methanol–chloroform (2:1, *v*/*v*) solution containing internal standards (N-dodecanoylsphingosine, N-dodecanoylglucosylsphingosine, N-dodecanoylsphingosylphosphorylcholine, and C17-sphinganine, 0.2 nmol each, from Avanti Polar Lipids) was added to the myocardial samples. The samples were extracted at 48 °C overnight and cooled. Then, 75 µL of 1 M KOH in methanol was added to saponify phospholipids and prevent their possible interference in the detection of sphingolipids, and the mixture was incubated for 2 h at 37 °C. Following the addition of 75 µL of 1 M acetic acid, the samples were evaporated to dryness, and stored at −20 °C until analysis. Before analysis, 150 µL of methanol was added to the samples. Then, the samples were centrifuged at 13,000× *g* for 5 min, and 130 µL of the supernatant was transferred to a new vial and injected.

#### 2.6.3. UPLC Coupled to HRMS Analysis

Ultra Performance Liquid chromatography (UPLC) coupled to high-resolution mass spectrometry (HRMS) analysis was performed using an Acquity ultra-high-performance liquid chromatography (UHPLC) system (Waters, USA) connected to a Time of Flight (LCT Premier XE) Detector. The full-scan spectra from 50 to 1800 Da were acquired, and individual spectra were summed to produce data points each being 0.2 s. Mass accuracy at a resolving power of 10,000 and reproducibility were maintained using an independent reference spray via LockSpray interference. 

Lipid extracts were injected onto an Acquity UPLC BEH C8 column (1.7 µm particle size, 100 mm × 2.1 mm, Waters Ireland) at a flow rate of 0.3 mL/min and a column temperature of 30 °C. The mobile phases were water with 2 mM of ammonium formate and 0.2% of formic acid (A) and methanol with 2 mM of ammonium formate and 0.2% of formic acid (B).The UPLC conditions were programmed as follows: 0.0 min_80% B; 3 min_90% B (linear gradient); 6 min_90% B (isocratic); 15 min_99% B (linear gradient); 18 min_99% B (isocratic); 20 min_80% B (linear gradient); 22 min_80% B (isocratic). 

Positive identification of compounds was based on the accurate mass measurement and its LC retention time, compared with that of a standard (<2%). Selected ions for SLs and glycerophospholipids correspond to [M + H]^+^, whereas ammonia adduct were used for neutral lipids (Table S1). The ceramide (Cer) standards used were N-palmitoyl-sphingosine, N-stearoyl-sphingosine, N-lignoceroyl-sphingosine and N-nervonoyl-sphingosine. The sphingomyelin (SM) standards used were N-palmitoylsphingosyl phosphorylcholine, egg SMs (predominant C16:0SM) and brain SMs (C18:0SM, C24:0SM and C24:1SM in known percentages). The glucosylceramide standard used was N-palmitoylglucosylsphingosine. The lactosylceramide standard used was N-palmitoyl-lactosylsphingosine. The diacylphospholipid standards used were 1,2-dipalmitoyl-snglycero- 3-phosphocholine, 1-palmitoyl-2-oleoyl-sn-glycero- 3-phosphocholine, 1-palmitoyl-2-oleoyl-sn-glycero-3-phosphoethanolamine and 1-palmitoyl-2-oleoyl-sn-glycero- 3-phospho-L-serine. The lysophospholipid standards used were 1-stearoyl-2-hydroxy-sn-glycero-3-phosphocholine, 1- oleoyl-2-hydroxy-sn-glycero-3-phosphoethanolamine and 1-oleoyl-2-hydroxy-sn-glycero-3-phospho-L-serine. The triacylglycerol standard used was 1,2,3-tri-(9Z-octadecenoyl)-glycerol. The cholesteryl ester standard used was cholesteryl cis-9-octadecenoate. When authentic standards were not available, identification was achieved based on their accurate mass measurement, elemental composition, calculated mass, error, double-bond equivalents and retention times. Quantification was carried out using the extracted ion chromatogram of each compound, obtained with a mass chromatogram absolute window value of 0.05 Da. As an example, representative ion chromatograms for sphingolipids (Figure S1), glycerophospholipids (Figures S2 and S3) and neutral lipids (Figure S4) of the right, left and infarcted left ventricle from the same patient are shown. The linear dynamic range was determined by injecting mixtures of internal and natural standards indicated above. If values were above the linear dynamic range, samples were diluted and analyzed again. Since standards for all identified lipids were not available, the amounts of lipids are given as pmol equivalents relative to each specific standard (Table S1). 

Sphingolipids (Cer: ceramide; SM: sphingomyelin; CDH: Ceramide dihexoside; HexCer: hexosylceramides), glycerophospholipids (PC: phosphatidylcholine; LPC: lysophosphatidylcholine; PE: phosphatidylethanolamine; LPE: lysophosphatidylethanolamine; PS: phosphatidylserine; LPS: lysophosphatidylserine, PG: phosphatidylglycerol), and neutral lipids (TAG: triacylglycerol; CE: cholesteryl esters; free cholesterol: FC) were detected. All glycerophospholipid and neutral lipid species were annotated using the “lipid subclass” and “C followed by the total fatty acid (FA) chain length:total number of unsaturated bonds” (e.g., PC (32:2)).

### 2.7. Statistical Analysis

Variables were compared two by two among the 3 groups (RV, LV and LV INF) using Student’s *t*-test or a Mann–Whitney U test for paired samples (myocardial samples were from the same patient), respectively, depending on whether the variables were normal. Normality was tested with a Shapiro–Wilk and Lilliefors test. *p* values were adjusted with Bonferroni’s correction, we performed 3 comparisons for each variable. 

The correlations between variables were studied in each group using Pearson’s correlation. *p* values were adjusted with Bonferroni’s correction because we tested the correlation among all lipidomic and biophysical variables. *p* < 0.05 was considered statistically significant. 

Lipidomic variables whose differences were significant in some of the study groups were represented in a heatmap. Each variable was represented as a logarithm of the quotient of the group mean and the mean value in all groups.

Statistical power was calculated afterwards as few lipidomic studies in human hearts have been performed until now and it was difficult to estimate the effect size in some lipidomic variables. Assuming the differences obtained in the significant variables as the real effect size, we obtained a range of values of statistical power between 0.64 and 0.98 in differential analysis and a range between 0.79 and 1 in correlation analysis. 

All data analyses were performed using R 4.0.2 software [29]. 

## 3. Results

### 3.1. Identification of the Main FTIR Bands in the Human Heart 

**Figure 1:**
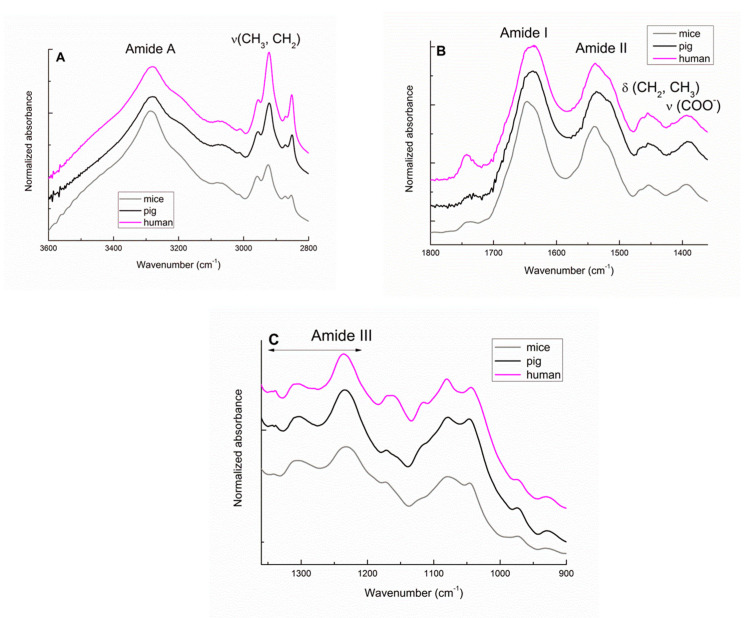
Averaged Fourier transform infrared spectroscopy (FTIR) spectra of the right ventricles from the mice, pig, and human samples. Line graphs showing the 3600–2800 cm^−1^ (**A**), 1800–1350 cm^−1^ (**B**), and 1350–900 cm^−1^ (**C**) spectral regions of mice, pig, and human right ventricles.

To compare the spectral signature of the human heart with previous data collected from animal hearts [4,5], the average FTIR spectra of mice, pig, and human right ventricles were superimposed (Figure 1). Table S2 summarizes the different FTIR absorption bands detected in human compared to pig and mice right ventricles, as well as their assignments, according to previously published data in transmission or ATR mode [5,6,30,31,32,33,34,35,36,37]. The major absorption bands in these spectra were the amide A, the amide I, and the amide II, which are mainly associated with proteins in freeze-dried tissues. Since Amide A is located in the large wave number zone where FTIR-ATR is not the more appropriate mode, the intensity of this mode has not been used for further semi-quantitative analysis. The primary ventricular proteins are cardiomyocyte myofibrillar (myosin, α-actin) and sarcoplasmic proteins and the structural proteins of the extracellular matrix (ECM), i.e., fibrillar collagens I and III. Among these different vibrations, the 1338 cm^−1^ band (wagging of the proline side chain) [6,25,36] corresponds to a specific signature of the structural proteins of the ECM since it is the only one that does not overlap with absorption or other components, e.g., DNA, lipids, or proteoglycans. As shown in Figure 1, the spectral signatures of the structural proteins of the ECM and the myofibers from mice, pigs and humans are very similar.

The complex FTIR spectra of the ventricles also include lipids with their classic markers at 2800–3000 cm^−1^ (especially the CH_2_ stretching of long hydrocarbon chains) (Figure 1A) and at 1475–1450 cm^−1^ (CH_2_ scissoring and CH3 bending) (Figure 1B). These lipids are mainly phospholipids of the plasmatic membranes, confirmed by the presence of the C=O stretching of ester groups, the CO-O-C and PO_2_^−^ stretching bands, triglycerides, cholesteryl esters, free cholesterol and free fatty acids (C=O stretching at 1712 cm^−1^ and COO^-^ stretching at 1392 cm^−1^). Unsaturated lipids are specifically marked by the 3013 cm^−1^ band.

Where the band position of the lipids is quasi-identical among the three species, the lipid vibrational response is more intense in human ventricles (for both the ν(CH2) and ν(C=O) bands) than in pig and mice ventricles.

Finally, as shown in Figure 1C, other ventricular components contributed to this complex vibrational response including DNA, specifically at 974 cm^−1^ [37]; proteoglycans (contributing to the 1079 cm^−1^ band and the overlapping 1226 cm^−1^ band); glycogen; and other polysaccharides (1200–1000 cm^−1^). The composite (1240–1100 cm^−1^) zone is subjected to fine differences due to disparities in the nature or proportion of these components among the three compared species.

### 3.2. Characterization of the Human Heart Lipidome

UPLC–HRMS analysis electrospray ionization mass spectrometry revealed 44 species of sphingolipids, 60 species of glycerophospholipids, and 60 species of neutral lipids in the human heart. The relative amount of each lipid in the whole family was expressed as the percentage of each lipid area compared to the total number of lipids of the same family in the right and left ventricles (Figures S5–S7). 

The relative abundance of the species present for each particular lipid among the family of sphingolipids in the human heart is also shown (Figure S5A–E). The most abundant sphingolipid in the human heart is sphingomyelin d18:1 (SM, 88.53%), particularly the species with long fatty acid chains (from 14 to 18 C) (Figure S5A). Minor species include ceramide d18:1 (Cer, 6.94%) (Figure S5B), followed by dihydrosphingomyelin d18:0 (dhSM, 3.56%) (Figure S5C), hexosylceramide (d18:1) (HexCer, 0.58%) (Figure S5D) and ceramide dihexoside (18:1) (CDH, 0.39%) (Figure S5E). CDH (d18:14:0) was significantly higher in LV than in RV (p = 0.045) (Figure S5E).

The most abundant glycerophospholipids in human cardiac tissue are phosphatidylcholine (PC, 75.24%), mainly species with long fatty acid (FA) chains (from 32 to 38 C) (Figure S6A); followed by phosphatidylserine (PS, 8.43%) (Figure S6B); lyso-phosphatidylcholine (LPC, 6.62%) (Figure S6C), phosphatidylethanolamine (PE, 4.96%) (Figure S6D), lyso-phosphatidylethanolamine (LPE, 2.88%) (Figure S6E), lyso- phosphatidylserine (LPS, 1.12%) (Figure S6F) and phosphatidylglycerol (PG, 0.76%) (Figure S6G).

Finally, as shown in Figure S7, the neutral lipids in the human hearts contain a high percentage of triglycerides (TAG, 97.06%) (Figure S7A). Several TAG species with longer unsaturated chains of fatty acids (FAs), decreased in the LV compared to the RV. These TAG species were C53:2 (*p* = 0.041), C54:1 (*p* = 0.036), and C54:2 (*p* = 0.033). The decrease in TAG in human LV compared to RV was previously reported in a porcine model [5] and likely reflects a higher effort and more energetic consumption of LV. Most of the cholesterol was found in the form of free cholesterol (FC, 2.03%) (Figure S7B), with a minor proportion found as cholesteryl esters (CE, 0.90%) (Figure S7C). There were no statistically significant differences in FC or CE between RV and LV. 

### 3.3. Identification of the Alterations of the FTIR Bands Related to Myofibers and Structural Extracellular Matrix Proteins in Human LV INF compared to LV and RV

Table S3 shows a comparison of the IR bands between RVs, LVs and LV INFs of the human hearts compiled from IR spectra. Specific assignments of these bands were performed according to the literature [5,6,30,31,32,33,34,35,36,37]. Most of the IR bands previously described in murine and porcine cardiac control tissues are also present in the RV, LV, and LV INF of human hearts. 

**Figure 2:**
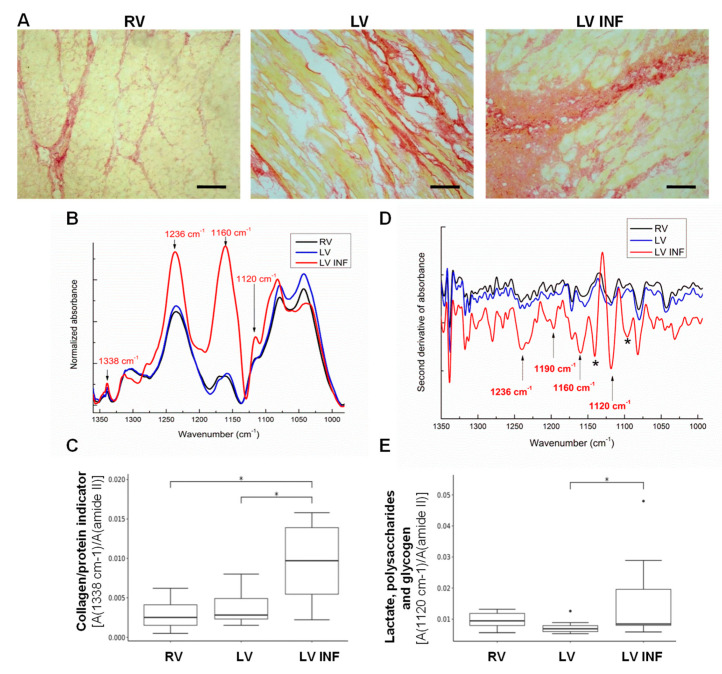
Immunohistochemical and FTIR analyses of fibrosis in human ventricle samples. Sirius Red Staining was used to distinguish the collagen (in red) in the right ventricle (RV), left ventricle (LV) and infarcted left ventricle (LV INF) samples. Scale Bar: 20 µm (**A**). Line graphs showing the 1350–1000 cm^−1^ averaged FTIR spectra of RV, LV, and LV INF (**B**). Boxplot analysis of the FTIR collagen/protein indicator (1338 cm^−1^/amide II) in the three groups (**C**). Line graphs showing the average second derivative spectra in the 1370–1000 cm^−1^ region (**D**). Arrows indicate the bands altered in the LV INF samples. Asterisks indicate the occurrence of new absorption bands in the LV INF samples. Boxplots showing lactate, polysaccharide, and glycogen/protein indicator (1220 cm^−1^/amide II) in the three groups (*n* = 9/group) (**E**). * *p* < 0.05. RV: right ventricle, LV: left ventricle, LV INF: infarcted left ventricle.

In agreement with the deep remodeling of infarcted tissue assessed by immunohistochemistry (Figure 2A), FTIR analyses revealed the profound alteration of the IR band pattern at 1300–860 cm^−1^ in the LV INF compared to the LV or RV samples (Figure 2B). A distinct feature of the collagen-specific absorption band at 1338 cm^−1^ was found in the human LV INF sample but not in the RV or LV samples. The indicator collagen/amide II (1338 cm^−1^/1540 cm^−1^), which reflects the content of structural proteins related to the total proteins, was significantly increased in LV INF compared to LV (*p* = 0.023) and RV (*p* = 0.023) (Figure 2C). The second derivative FTIR spectra (which allows increased resolution) showed the exclusive presence of certain IR bands in LV INF at 1140 and 1095 cm^−1^ (Figure 2D, asterisks) in the ν(C-O-C)/ν(C-OH) absorption zone, indicating deep alterations in oligosaccharides and also in glycolipids, phospholipids, and nucleic acids in the infarcted area. In addition, intense absorptions at 1120 cm^−1^ (characteristic of ν(C-O) in lactate, polysaccharides, and glycogen), 1236 cm^−1^ (amide III), and 1160 cm^−1^ (hydroxyproline residues, collagen specific) were detected in human LV INF compared to LV and RV (Figure 2B,D, arrows). The increase of 1236 and 1160 cm^−1^ band absorptions could be due to augmented collagen deposition in the infarcted zone, while that of 1120 cm^−1^ is associated with elevated levels of lactate and/or polysaccharides in this zone (*p* = 0.047) (Figure 2E).

### 3.4. Identification of the Alterations in the FTIR Bands Corresponding to DNA Response in Human LV INF Compared to LV and RV

**Figure 3:**
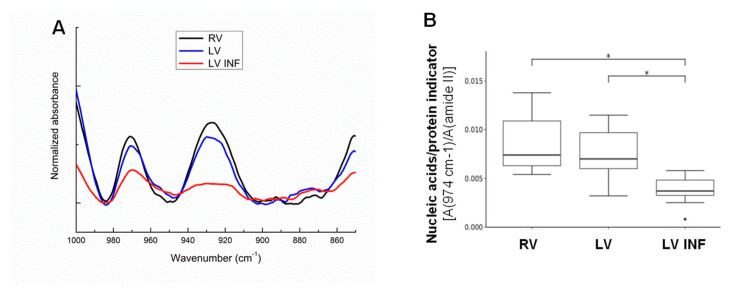
FTIR analysis of the nucleic acids in the human ventricle samples. Line graphs showing the 1000–850 cm^−1^ average FTIR spectra corresponding to the signal of nucleic acids in the RV, LV, and LV INF samples (**A**). Boxplot analysis of nucleic acid/protein FITR indicator (974 cm^−1^/amide II) (**B**) in the three groups (*n* = 9/group). * *p* < 0.05. RV: right ventricle, LV: left ventricle, LV INF: infarcted left ventricle.

As reported in Figure 3A, FTIR spectra corresponding to 1000–850 cm^−1^ where the DNA/RNA response prevails, showed differences between the three myocardial samples, which were quantified by the nucleic acid/protein indicator (band area ratio 974 cm^−1^/1540 cm^−1^, Figure 3B). There was a significant decrease in the DNA/protein ratio from the right to left ischemic ventricles, reaching significance in LV INF vs. LV (*p* = 0.0056) and RV (*p* = 0.0061) (Figure 3B).

This evolution is consistent with previous FTIR imaging results on infarcted myocardium, indicating that nucleic acids are widespread in a normal myocardium but destroyed in an infarcted myocardium [38]. All changes revealed the profound remodeling of the ECM in human infarcted tissue.

### 3.5. Study of the Hydric Response of Human RV, LV and LV INF

Representative DSC thermograms (normalized to the initial mass) of fresh human ventricles corresponding to cooling from 20 to −100 °C and successive heating from −100 to 25 °C are reported in Figure S8A. The cooling thermograms are characterized by an intense exothermic peak corresponding to the crystallization of free water (namely, water not bound to hydrophilic components and able to form ice). The onset temperature of the peak (Ton) is attributed to the transition temperature of this thermal event. The heating thermograms are characterized by an endothermic peak corresponding to the melting of previously frozen water. Weak and multiple thermal events are detectable in the (50–85 °C) zone and attributed to the denaturation of cardiac muscle proteins, including myosin, sarcoplasmic proteins, collagen and actin as already observed in a previous study [4]. There is a significant decrease in the temperature of water crystallization Tc for LV INF compared to LV (*p* = 0.044) (Figure S8B). A similar trend is observed for the ice melting recorded in the heating scans. The increase in aqueous salt concentrations could explain such a depression of the melting/crystallization temperature. However, the typical signature of aqueous salts, namely the eutectic phase transition at lower temperature, was not detected in the DSC thermograms of myocardial samples. Since water crystallization and ice melting are related to the pore size in porous [39] and biological materials [40], the differences between the infarcted and non-infarcted zones can be interpreted as changes in the architecture associated with a decrease of the pore’s nominal radius. Cardiac remodeling, including the replacement of myofibers by collagen fibers marking a difference in the tissue architecture (pore size), could explain this peculiar thermal behavior. 

### 3.6. Study of the Associations between FITR Variables in Human RV, LV and LV INF

**Figure 4:**
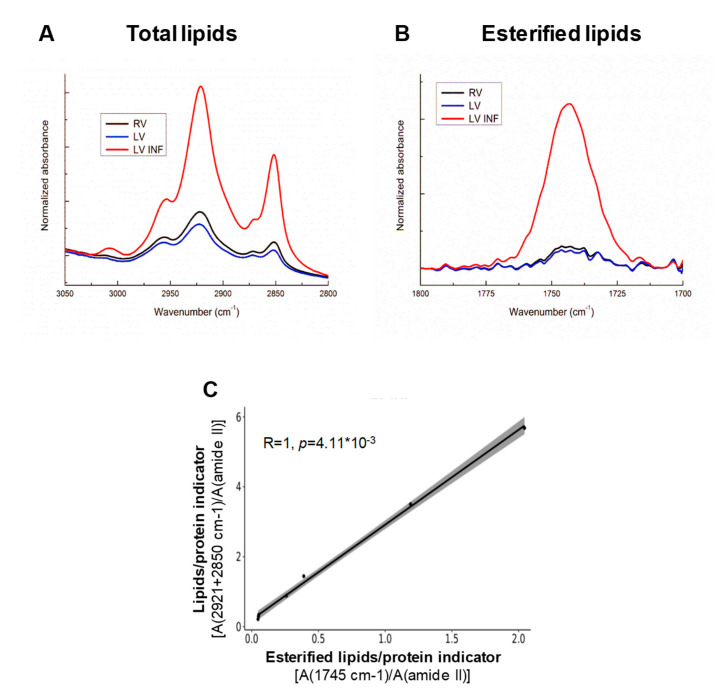
FTIR analysis of the total and esterified lipids in the human ventricle samples. Line graphs showing the 3050–2800 cm^−1^ (**A**) and 1800–1700 cm^−1^ (**B**) average FTIR spectra corresponding to the signals of the total and esterified lipids, respectively, in right ventricle (RV), left ventricle (LV), and infarcted left ventricle (LV INF). (**C**) Correlation analysis between the FTIR indicators of the total and esterified lipids in LV INF. The correlation was studied with Pearson’s correlation, and the *p*-values were adjusted with Bonferroni’s correction. RV: right ventricle, LV: left ventricle, LV INF: infarcted left ventricle.

The FTIR spectra show that the CH_2_ bands (2921 cm^−1^ and 2850 cm^−1^) in the (CH_2_, CH_3_) stretching zone (3000–2800 cm^−1^) are mainly associated with the lipid signature (Figure 4A) and the C=O stretching zones of the ester carbonyl groups of phospholipids, triglycerides, and cholesteryl esters (Figure 4B). There was an intensification of the lipid bands in the left infarcted ventricles, and a close correlation (R = 1, *p* = 8.7 × 10^−8^) between the total lipid (2921–2850 cm^−1^/1540 cm^−1^) and the esterified lipid (1745 cm^−1^/1540 cm^−1^) indicators (Figure 4C) in this zone. There were no correlations statistically significant between total and esterified lipids in RV or LV (R = 0.93, *p* = 1; R = 0.89, *p* = 1, respectively), suggesting that most of the lipids are esterified in the infarcted LV, but not in the RV or LV.

### 3.7. Identification of the Alterations in Sphingolipids, Glycerophospholipids and Neutral Lipids in Human LV INF Compared to LV and RV

**Figure 5:**
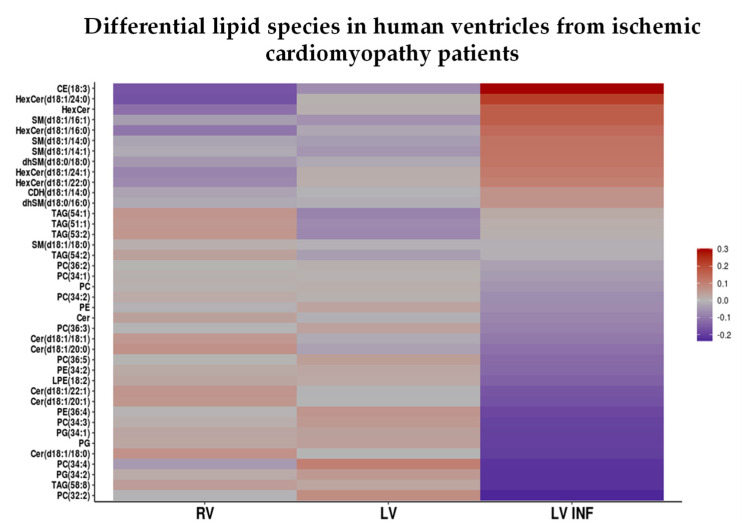
Lipidomic analysis of the human right ventricle, left ventricle and infarcted left ventricle. Heatmap of the nine human heart samples (in columns) based in the quantification of 40 differential lipid species (in rows) that exhibited statistically significant differences among right ventricle (RV), left ventricle (LV), and infarcted left ventricle (LV INF) samples. The heatmap colors represent a decimal logarithm of the quotient of the group mean and the mean value in all groups for each variable. Blue cells indicate values below the mean value of the variables in all groups, while red cells indicate values above the mean value of the variables in all groups. CE: cholesteryl esters, HexCer: hexosylceramide, SM: sphingomyelin, dhSM: dihydrosphingomyelin, CDH: ceramide dihexoside, TAG: triacylglycerols, PC: phosphatidylcholine, PE: phosphatidylethanolamine, LPE: lysophosphatidylethanolamine, Cer: ceramide, PG: phosphatidylglycerol. RV: right ventricle, LV: left ventricle, LV INF: infarcted left ventricle.

Lipidomic studies reflected deep lipid remodeling in the infarcted tissue. As visualized in the heatmap of Figure 5, cholesteryl ester and several sphingolipid species were significantly increased in LV INF compared to RV or LV, while most of the glycerophospholipid species were significantly decreased in the infarcted cardiac tissue. As shown in Table 2, SM 18:1 (14:0 and 14:1) and 16:1, dhSM 18:0 (16:0 and 18:0), and HexCer 18:1 species (16:0, 22:0, 24:0, 24:1) were significantly increased, while Cer 18:1 (18:0, 20:0, 20:1, 22:1 and 24:1) species were significantly decreased in the LV infarcted tissue (Table 2). There were no significant differences in CDH or the rest of the sphingolipid species between LV INF and LV or RV (Table S4). Like Cer, the main glycerophospholipids, including PE (34:2, 36:4), LPE (18:2), PC (32:2, 34:1, 34:2, 34:3, 34:4, 36:2, 36:3, 36:5), and PG (34:1, 34:2), were significantly decreased in human infarcted tissue (Table 3). There were no significant differences in the PS, LPS, or LPC and the rest of glycerophospholipid species between LV INF and LV or RV (Table S5). Finally, among the neutral lipids, only cholesteryl esters were found to be significantly increased in the infarcted tissue (18:3) (Table 4). There were no differences in the highly abundant TAG species or minor FC species between LV INF and LV or RV (Table S6).

### 3.8. Study of the Association between FTIR and Lipidomic Variables in RV, LV and LV INF

**Figure 6:**
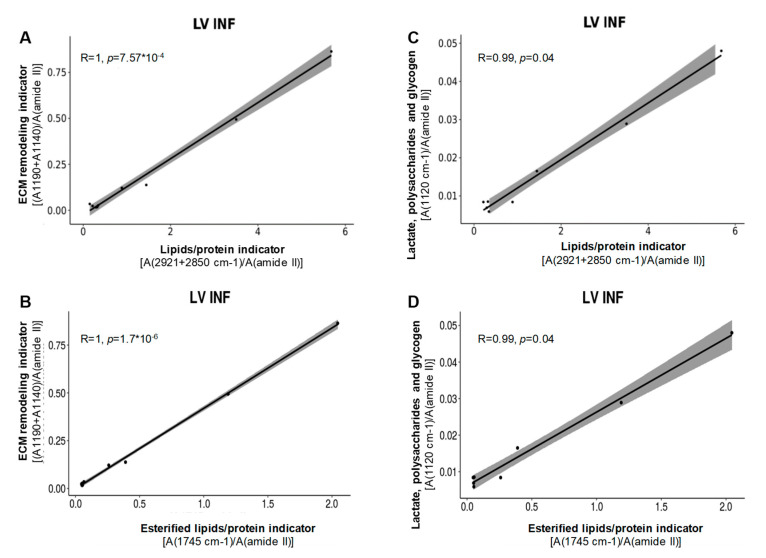
Correlation analysis of FTIR indicators of cardiac remodeling and lipids in the human heart. Correlation between the extracellular matrix (ECM) remodeling indicator and both total lipids (**A**) and esterified lipids (**B**), as well as between lactate, polysaccharide, and glycogen and both total lipids (**C**) and esterified lipids (**D**), in human infarcted left ventricle. The correlation was studied with Pearson’s correlation, and *p* values were adjusted with Bonferroni’s correction. LV INF: infarcted left ventricle.

As shown in Figure 6, biophysical studies revealed that ECM remodeling was closely associated with lipid indicators, such as total lipids (2921–2850 cm^−1^) (R = 1, *p* = 1.6 × 10^−8^) (Figure 6A) and esterified lipids (1745 cm^−1^/amide II) (R = 0.99, *p* = 9.3 × 10^−7^) (Figure 6B) specifically in LV INF. In addition, other extracellular matrix markers such as lactate, polysaccharides, and glycogen, were also closely associated with total lipids (R = 1, *p* = 3.6 × 10^−11^) (Figure 6C) and esterified lipids (R = 0.99, *p* = 9.1 × 10^−7^) (Figure 6D) in the cardiac infarcted tissue. There was no significant correlation between lipid and ECM remodeling FITR indicators in human RV (R = 0.54, *p* = 1) or LV (R = 0.68, *p* = 1).

## 4. Discussion

In this work, we used the FTIR spectrum to illustrate the overall variety of changes in proteins, nucleic acids, and lipids that occur in human cardiac infarcted tissue. FTIR spectroscopy is very sensitive to variations in cell metabolism, making it highly suitable for defining the structure in the infarcted areas where the tissue has undergone strong cardiometabolic alterations primarily associated with the process of ischemia [41]. 

The spectral signatures of the structural proteins of ECM and the myofibers in the human heart are similar to those previously reported in murine [4] and porcine hearts [5]. One of the most significant alterations observed in the FTIR spectra of human LV INF vs. LV or RV was a strong increase in the 1338 cm^−1^ band, which has been shown to be specific to collagen [6,25,36]. The utility of this particular FTIR band to monitor the evolution of cardiac pathology and therapeutics was previously validated in a rat MI model [6]. Another FTIR band that is deeply altered in the context of MI is the 974 cm^−1^ band assigned to nucleic acids that, as expected, suffered a strong decline in LV INF compared to LV and RV. These changes reflected a profound remodeling of the LV INF accompanying cardiomyocyte death in the context of myocardial infarction and highlighted the potential of infrared markers to trace the incidence of ischemia in cardiac remodeling.

The spectral signatures of lipids are also quasi-identical in the human heart to those in previous murine [4] and pig [5] hearts. The vibrational response of the lipids is much more intense (for both the ν(CH2) and ν(C=O) bands, corresponding to the total lipids and ester carbonyl groups of PL and neutral lipids) in human than in murine or porcine ventricles. Lipidomic analysis of the human heart revealed that the main lipids present are SM (d18:1) (88% of the total sphingolipids), PC (75% of the total glycerophospholipids) and TAGs (97% of the total neutral lipids). There are few detailed lipidomic studies of ventricles and almost none on humans, likely due to the difficulty of obtaining human heart samples. We found several species belonging to the sphingolipid, glycerophospholipid, and neutral lipid families that were altered in LV INF but not in RV and LV. Most of the glycerophospholipids decreased, while most of the sphingolipids and, specific species of cholesteryl esters increased in the infarcted human cardiac tissue. 

Between sphingolipids, the levels of several species of SM, dhSM, and HexCer were increased in LV INF compared to LV or RV. The increase in the SM content of infarcted tissue found in the present work seems to be coherent with the decreased sphingomyelinase (SMase) activity previously described [42]. The increase in SM that we found in humans differs from the results showing lower SM levels in the infarcted brains of in vivo models of ischemic stroke [43]. The cleavage of SM by acid or neutral SMase results in the liberation of ceramide, a sphingolipid that acts as an intracellular messenger regulating the activity of kinases, phosphatases, and transcription factors. Ceramides were reported to be upregulated in the ischemic zones of the heart in several in vivo models of ischemia/reperfusion [13,44,45]. This increase in ceramides after ischemia reperfusion appears to be transient and linked with reperfusion injury, as shown in a model of ischemic-reperfused myocardium in rats [42]. It has also been shown that the post-MI increase in cardiac ceramide is not caused by increased SMase activity but instead by decreased ceramidase activity. In addition, it has been recently demonstrated that a transient increase in acid ceramidase is sufficient to induce cardioprotection post-MI [46]. Ceramide is utilized for the synthesis of other bioactive sphingolipids, including sphingosine-1-phosphate, sphingosine, and the glycosphingolipids lactosylceramide and hexosylceramide (HexCer). Here, several species of HexCer were found accumulated in human infarcted hearts. Previous studies have reported the accumulation of HexCer in several organs from in vivo models of aging [47,48]. Clinically, increased circulating levels of both SM and Cer have been correlated with an increased risk of coronary artery disease [49,50]. 

Between glycerophospholipids, we found that the levels of the main PL, phosphatidylcholine (PC), and other less abundant PLs, such as phosphatidylethanolamine (PE) and phosphatidylglycerol (PG), were significantly decreased in LV INF compared to LV. These results are in agreement with previous studies performed in in vivo MI models [16,17] showing a strong PL decay caused by ischemia-induced phospholipolysis of the cardiomyocyte membranes. Increased phospholipolysis can be caused, among other factors, by the upregulatory effect of hypoxia on phospholipase activity [51,52]. Unbalanced levels of anionic phospholipids such as PG seem to have harmful consequences for mitochondrial morphology and function. PG plays a pivotal role in the formation of cardiolipin, which is essential in the control of mitochondrial inflammation and oxidative stress [53,54], and respiratory activity [54]. In this study, we found a significant loss of PG that could, indeed, contribute to a serious decrease in mitochondrial oxidative metabolism in infarcted tissue. The strong decrease in mitochondrial respiratory activity combined with acute phospholipolysis, which actively generates fatty acids [16,17], could be a determinant for increased FA esterification. An additional source of esterified lipids in the infarcted tissue is circulating lipoproteins. Several groups, including ours, have reported the upregulatory effect of hypoxia/ischemia on lipoprotein receptors such as Very low-density lipoprotein receptor VLDLR and Low-density lipoprotein receptor related-protein 1 LRP1, which bind and internalize lipoproteins such as Very low-density lipoprotein (VLDL) and Low-density lipoprotein (LDL) (lipoproteins highly enriched in cholesteryl esters) [11,12,13,55,56]. Here, the close correlation between esterified lipids and remodeling in the human infarcted tissue pointed to FTIR lipid indicators as potential biomarkers of remodeling, at least in the context of ischemia. Previous studies from our group demonstrated that the levels of intracellular esterified lipids in cardiomyocytes determine the structural and physical characteristics of secreted tropoelastin through an increase in cathepsin S mature protein levels [57]. The intracellular esterified lipids stored in lipid droplets are the subject of an intense debate over their potential beneficial/harmful effects on cell functionality [58]. The differential implications of triglyceride and cholesteryl ester proportions in these lipid droplets are also under discussion [59].

## 5. Conclusions

As a general conclusion, FTIR studies showed that human cardiac infarcted tissue suffers deep alterations in proteins, nucleic acids, and—especially—lipids. Liquid chromatography coupled to high-resolution mass spectrometry (LC–HRMS) revealed the strong lipid remodeling that results in reduced levels of PC, PE, LPE and PG and increased levels of SM, HexCer and cholesteryl esters in human cardiac infarcted tissue. We found a strong and positive association between esterified lipids and adverse cardiac remodeling in the context of human cardiac remodeling post-MI. The specific conclusions are that (i) there are deep alterations of the FTIR bands related to myofibers and structural extracellular matrix proteins in human LV INF compared to LV and RV, (ii) there are strong differences in the FTIR spectra corresponding to DNA responses in human LV INF compared to RV and LV, (iii) there is a differential hydric response of human LV INF compared to RV and LV, (iv) FTIR combined with lipidomic studies showed a strong intensification of lipids, particularly esterified lipids concomitantly with deep phospholipid remodeling in human infarcted hearts, and (v) esterified lipids are closely related with adverse cardiac remodeling in human infarcted heart. Thus, we have shown that FTIR lipid indicators are potential biomarkers of cardiac remodeling and validated certain lipid species as crucial in human pathological ventricular remodeling post-MI.

## Figures and Tables

**Table 1:** Clinical and echocardiographic characteristics from whom explanted ischemic hearts were obtained.

	ICM (*n* = 9)
Age (years)	57.67 ± 12.02
Gender male (%)	78
Prior Hypertension (%)	44
Diabetes mellitus (%)	11
Dislipemia (%)	75
Perfusion abnormalities * (%)	72
Echo-Doppler study	
Ejection fraction (%)	29.88 ± 8.90
Intraventricular septum in diastole (mm)	11.63 ± 4.63
Left ventricular posterior wall in diastole (mm)	9.53 ± 0.75
Left ventricular end-diastolic diameter (mm)	60.38 ± 14.15
Left ventricular end-systolic diameter (mm)	56.25 ± 2.79
Treatment (%)	
Diuretics	89
Angiotensin-converting enzyme inhibitors	86
β-blockers	48
Aldosteron antagonists	71
Digoxin	49
Statins	82

ICM, ischemic cardiomyopathy; * the patients with perfusion abnormalities were considered those subjected to coronary interventions (i.e. by-pass, angioplasty, stents and others).

**Table 2:** Sphingolipid species with differential concentrations in the infarcted left ventricle compared to left ventricle or right ventricle from human explanted ischemic hearts.

Variable	RV	LV	LV INF	*p* vs. LV	*p* vs. RV
SM(d18:1/14:0)	308.5 ± 77.4	309.5 ± 95.9	430.5 ± 95.3	0.024	0.055
SM(d18:1/14:1)	3 ± 1.2	2.8 ± 1	3.9 ± 1.4	0.029	0.096
SM(d18:1/16:1)	164 ± 39.5	160.1 ± 56.8	258.8 ± 105.8	0.029	0.041
SM(d18:1/18:0)	1199.2 ± 156.3	1138.7 ± 128.5	1086.2 ± 142.7	0.485	0.038
Total Cer	753 ± 261.6	592.7 ± 240.1	549.5 ± 179.1	0.932	0.044
Cer(d18:1/18:0)	30.4 ± 13.5	22.9 ± 14.5	15 ± 4.9	0.115	0.029
Cer(d18:1/18:1)	7.2 ± 2.3	5.2 ± 2	4.6 ± 1.1	0.456	0.006
Cer(d18:1/20:0)	14.5 ± 3.6	11.1 ± 4	9.6 ± 2.8	0.457	0.01
Cer(d18:1/20:1)	4.4 ± 0.8	3.4 ± 1.3	2.6 ± 0.6	0.13	0.006
Cer(d18:1/22:1)	28.7 ± 22.9	20.2 ± 16.1	17.5 ± 20.1	0.804	0.018
dhSM(d18:0/16:0)	179.4 ± 41.3	207.5 ± 91.7	226.4 ± 65	0.931	0.029
dhSM(d18:1/18:0)	46.3 ± 13.4	49.5 ± 23.3	64.5 ± 16.3	0.174	0.041
Total HexCer	48.1 ± 26.6	63.9 ± 31.2	91.3 ± 36.6	0.114	0.006
HexCer(d18:1/16:0)	2.3 ± 1.5	3.2 ± 1.7	4.4 ± 3.1	0.474	0.023
HexCer(d18:1/22:0)	10.9 ± 5.8	16.1 ± 13.2	16 ± 5.6	1	0.012
HexCer(d18:1/24:0)	19.6 ± 10.4	25.3 ± 13.6	44.3 ± 25.1	0.127	0.018
HexCer(d18:1/24:1)	15.5 ± 10.5	20 ± 5.6	26.7 ± 13.5	0.102	0.006

Data are expressed as mean ± SD, *n* = 9 (RV, LV and LV INF). SM: sphingomyelin, Cer: Ceramide, dhSM: dihydrosphingomyelin, HexCer: Hexosylceramide, SD: standard deviation, RV: right ventricle, LV: left ventricle, LV INF: infarcted left ventricle.

**Table 3:** Glycerophospholipid species with differential concentrations in the infarcted left ventricle compared to left ventricle or right ventricle from human explanted ischemic hearts.

Variable	RV	LV	LV INF	*p* vs. LV	*p* vs. RV
Total PC	54,624.9 ± 11408.6	56,737 ± 5670.8	48,392.9 ± 4893.7	0.012	0.335
PC(32:2)	342.5 ± 146.2	389.6 ± 115.9	221.1 ± 103.4	0.029	0.102
PC(34:1)	14,602.8 ± 2506.6	15,143.9 ± 1769.8	13,505.1 ± 1492.7	0.029	0.556
PC(34:2)	13,397 ± 2572.8	13,827.1 ± 1685	11,574.9 ± 1501.8	0.006	0.232
PC(34:3)	120.7 ± 54.3	133.2 ± 46.3	71.9 ± 21.8	0.012	0.049
PC(34:4)	100.7 ± 52.6	133.8 ± 67.5	64.7 ± 34.1	0.041	0.077
PC(36:2)	4753.8 ± 1047.4	4902.2 ± 444.3	4439.5 ± 328.1	0.025	0.698
PC(36:3)	3650 ± 1035	3884.2 ± 936.2	2866.5 ± 856.6	0.003	0.122
PC(36:5)	391.6 ± 437.5	376.8 ± 327.3	236.4 ± 269.4	0.041	0.069
Total PE	3502.5 ± 1052.4	3840.7 ± 914.1	2986.4 ± 769	0.004	0.285
PE(34:2)	203.1 ± 65.5	205.2 ± 47	144.1 ± 37.9	0.008	0.028
PE(36:4)	1014.5 ± 300.8	1309.3 ± 502.6	629.4 ± 269.1	0.011	0.036
LPE(18:2)	144.7 ± 60.3	142.7 ± 43.4	95 ± 30.3	0.006	0.052
Total PG	538.9 ± 135.4	581.8 ± 188.7	329.2 ± 152.5	0.045	0.041
PG(34:1)	483 ± 121.9	523 ± 176.8	300.9 ± 139.4	0.052	0.042
PG(34:2)	55.9 ± 20.6	58.8 ± 19.1	28.3 ± 14.9	0.028	0.047

Data are expressed as mean ± SD, *n* = 9 (RV, LV and LV INF). PC: phosphatidylcholine, PE:phosphatidylethanolamine, LPE: lysophosphatidylethanolamine, PG: phosphatidylglycerol, SD: standard deviation, RV: right ventricle, LV: left ventricle, LV INF: infarcted left ventricle.

**Table 4:** Neutral lipid species with differential concentrations in the infarcted left ventricle compared to left ventricle or right ventricle from human explanted ischemic hearts.

Variable	RV	LV	LV INF	*p* vs. LV	*p* vs. RV
TAG(58:8)	2856 ± 1534.1	3364.8 ± 3160.3	1978.9 ± 1520.3	0.018	0.075
CE(18:3)	269.5 ± 248.3	352.3 ± 319.7	575.9 ± 583.5	0.239	0.018

Data are expressed as mean ± SD, *n* = 9 (RV, LV and LV INF). TAG: triacylglycerols, CE: cholesteryl esters, SD: standard deviation, RV: right ventricle, LV: left ventricle, LV INF: infarcted left ventricle.

## Limitations of This Study

The main limitation of this study is the low number of patients included due to the difficulty of (i) obtaining samples from ischemic cardiomyopathy patients and (ii) performing biophysical and lipidomic studies on an elevated number of series. However, great differences in some variables were found between ventricle samples, as well as strong correlations between variables. There was also a high correlation between ECM remodeling indicators and the total and esterified lipids in LV INF. Furthermore, we have obtained relatively high values of statistical power (between 0.64–0.98), despite the low number of samples, due to the big differences in some variables between the ventricles. Regardless, more studies are needed to confirm our results and provide higher statistical power. We also consider, as limitation of this study, the impossibility to characterize the infarcted right ventricle due to the low prevalence and diagnose of this entity. Finally, we could not assess the levels of cardiolipin, a crucial lipid in cardiovascular disease, due to the technical unavailability to perform this analysis.

## Supplementary Materials

The following are available online at https://www.mdpi.com/2218-273X/10/11/1471/s1, Figure S1: Representative ion chromatograms of ceramides and sphingomyelins detected in the right, left and infarcted left ventricle of the same patient; Figure S2: Representative ion chromatograms of phosphatidylcholines (PCs) and phosphatidylethanolamines (PEs) detected in the right, left and infarcted left ventricle of the same patient; Figure S3: Representative ion chromatograms of phosphatidylserines (PSs) and lysophospholipids (PEs) detected in the right, left and infarcted left ventricle of the same patient; Figure S4: Representative ion chromatograms of neutral lipids detected in the right, left and infarcted left ventricle of the same patient; Figure S5: Levels of sphingolipids species in right and left ventricles from human explanted ischemic hearts; Figure S6: Levels of glycerolipids species in right and left ventricles from human explanted ischemic hearts; Figure S7: Levels of neutral lipid species in right and left ventricles from human explanted ischemic hearts; Figure S8: Hydric response of the right, left and left infarcted ventricles from human explanted ischemic hearts; Table S1: Lipids identified in the myocardial samples; Table S2: FTIR band position and assignment in human, pig and mice right ventricle samples; Table S3: FTIR band position and assignment in right, left and left infarcted ventricles from human explanted ischemic hearts; Table S4: Levels of sphingolipids species (pmol equiv/mg prot) in right, left and left infarcted ventricles from human ischemic hearts; Table S5: Levels of glycerophospholipid species (pmol equiv/mg prot) in right, left and left infarcted ventricles from human ischemic hearts; Table S6: Levels of neutral lipid species (pmol equiv/mg prot) in right, left and left infarcted ventricles from human ischemic hearts.
