# Supplementary material for: Biophysical and Lipidomic Biomarkers of Cardiac Remodeling Post-Myocardial Infarction in Humans
Source: Biomolecules. 2020 Oct 22;10(11):1471. doi: 10.3390/biom10111471 (PMC7690619; doi:10.3390/biom10111471)
Supplement: Supplementary file 1 [file biomolecules-10-01471-s001.pdf]

SUPPLEMENTAL MATERIALS

Supplemental Tables

Table S1. Lipids identified in the myocardial samples.

SUB CLASS	SPECIE	IS REFERENCE	m/z	ION	RT (min)
[SP] Ceramides	**Cer**				
[SP] Ceramides	Cer (d18:1/14:0)	C12 Cer IS	510.489	[M+H]+	**6.32**
[SP] Ceramides	Cer (d18:1/16:0)	C12 Cer IS	538.520	[M+H]+	**7.69**
[SP] Ceramides	Cer (d18:1/16:1)	C12 Cer IS	536.504	[M+H]+	**6.69**
[SP] Ceramides	Cer (d18:1/18:0)	C12 Cer IS	566.551	[M+H]+	**9.13**
[SP] Ceramides	Cer (d18:1/18:1)	C12 Cer IS	564.536	[M+H]+	**8.19**
[SP] Ceramides	Cer (d18:1/20:0)	C12 Cer IS	594.583	[M+H]+	**10.54**
[SP] Ceramides	Cer (d18:1/20:1)	C12 Cer IS	592.567	[M+H]+	**9.63**
[SP] Ceramides	Cer (d18:1/22:0)	C12 Cer IS	622.614	[M+H]+	**11.79**
[SP] Ceramides	Cer (d18:1/22:1)	C12 Cer IS	620.598	[M+H]+	**11.01**
[SP] Ceramides	Cer (d18:1/24:0)	C12 Cer IS	650.645	[M+H]+	**12.91**
[SP] Ceramides	Cer (d18:1/24:1)	C12 Cer IS	648.629	[M+H]+	**11.95**
[SP] Ceramides	Cer (d18:1/24:2)	C12 Cer IS	646.614	[M+H]+	**11.16**
[SP] Sphingomyelin	**SM**				
[SP] Sphingomyelin	SM (d18:1/14:0)	C12 SM IS	675.544	[M+H]+	**6.13**
[SP] Sphingomyelin	SM (d18:1/14:1)	C12 SM IS	673.528	[M+H]+	**5.38**
[SP] Sphingomyelin	SM (d18:1/16:0)	C12 SM IS	703.575	[M+H]+	**7.38**
[SP] Sphingomyelin	SM (d18:1/16:1)	C12 SM IS	701.560	[M+H]+	**6.44**
[SP] Sphingomyelin	SM (d18:1/18:0)	C12 SM IS	731.607	[M+H]+	**8.82**
[SP] Sphingomyelin	SM (d18:1/18:1)	C12 SM IS	729.591	[M+H]+	**7.88**
[SP] Sphingomyelin	SM (d18:1/20:0)	C12 SM IS	759.638	[M+H]+	**10.23**
[SP] Sphingomyelin	SM (d18:1/20:1)	C12 SM IS	757.622	[M+H]+	**9.32**
[SP] Sphingomyelin	SM (d18:1/22:0)	C12 SM IS	787.669	[M+H]+	**11.51**
[SP] Sphingomyelin	SM (d18:1/22:1)	C12 SM IS	785.654	[M+H]+	**10.70**
[SP] Sphingomyelin	SM (d18:1/24:0)	C12 SM IS	815.701	[M+H]+	**12.70**
[SP] Sphingomyelin	SM (d18:1/24:1)	C12 SM IS	813.685	[M+H]+	**11.63**
[SP] Sphingomyelin	SM (d18:1/24:2)	C12 SM IS	811.669	[M+H]+	**10.82**
[SP] Sphingomyelin	SM (d18:1/24:3)	C12 SM IS	809.654	[M+H]+	**9.94**
[SP] Dihydrosphingomyelin	**dhSM**				
[SP] Dihydrosphingomyelin	dhSM (d18:0/14:0)	C12 SM IS	677.560	[M+H]+	**6.54**
[SP] Dihydrosphingomyelin	dhSM (d18:0/16:0)	C12 SM IS	705.591	[M+H]+	**7.91**

[SP] Dihydrosphingomyelin	dhSM (d18:0/18:0)	C12 SM IS	733.622	[M+H]+	**9.32**
[SP] Dihydrosphingomyelin	dhSM (d18:0/20:0)	C12 SM IS	761.654	[M+H]+	**10.70**
[SP] Dihydrosphingomyelin	dhSM (d18:0/22:0)	C12 SM IS	789.685	[M+H]+	**11.92**
[SP] Dihydrosphingomyelin	dhSM (d18:0/24:0)	C12 SM IS	817.716	[M+H]+	**13.04**
[SP] Hexosylceramide	**HexCer**				
[SP] Hexosylceramide	HexCer (d18:1/16:0)	C12 GlcCer IS	700.573	[M+H]+	**6.97**
[SP] Hexosylceramide	HexCer (d18:1/22:0)	C12 GlcCer IS	784.667	[M+H]+	**11.13**
[SP] Hexosylceramide	HexCer (d18:1/24:0)	C12 GlcCer IS	812.698	[M+H]+	**12.29**
[SP] Hexosylceramide	HexCer (d18:1/24:1)	C12 GlcCer IS	810.682	[M+H]+	**11.23**
[SP] Ceramide dihexoside	**CDH**				
[SP] Ceramide dihexoside	CDH (d18:1/14:0)	C12 GlcCer IS	834.594	[M+H]+	**5.60**
[SP] Ceramide dihexoside	CDH (d18:1/16:0)	C12 GlcCer IS	862.626	[M+H]+	**6.66**
[SP] Ceramide dihexoside	CDH (d18:1/18:0)	C12 GlcCer IS	890.657	[M+H]+	**8.10**
[SP] Ceramide dihexoside	CDH (d18:1/20:0)	C12 GlcCer IS	918.688	[M+H]+	**9.51**
[SP] Ceramide dihexoside	CDH (d18:1/22:0)	C12 GlcCer IS	946.719	[M+H]+	**10.85**
[SP] Ceramide dihexoside	CDH (d18:1/24:0)	C12 GlcCer IS	974.751	[M+H]+	**12.01**
[SP] Ceramide dihexoside	CDH (d18:1/24:1)	C12 GlcCer IS	972.735	[M+H]+	**10.98**
[GP] Phosphatidylcholines	**PC**				
[GP] Phosphatidylcholines	PC (32:0)	D31-PC(16:0/18:1) IS	734.570	[M+H]+	**8.41**
[GP] Phosphatidylcholines	PC (32:1)	D31-PC(16:0/18:1) IS	732.554	[M+H]+	**7.47**
[GP] Phosphatidylcholines	PC (32:2)	D31-PC(16:0/18:1) IS	730.539	[M+H]+	**6.54**
[GP] Phosphatidylcholines	PC (34:0)	D31-PC(16:0/18:1) IS	762.601	[M+H]+	**9.79**
[GP] Phosphatidylcholines	PC (34:1)	D31-PC(16:0/18:1) IS	760.586	[M+H]+	**8.85**
[GP] Phosphatidylcholines	PC (34:2)	D31-PC(16:0/18:1) IS	758.572	[M+H]+	**7.91**
[GP] Phosphatidylcholines	PC (34:3)	D31-PC(16:0/18:1) IS	756.556	[M+H]+	**7.32**
[GP] Phosphatidylcholines	PC (34:4)	D31-PC(16:0/18:1) IS	754.540	[M+H]+	**6.60**
[GP] Phosphatidylcholines	PC (36:0)	D31-PC(16:0/18:1) IS	790.633	[M+H]+	**11.01**
[GP] Phosphatidylcholines	PC (36:1)	D31-PC(16:0/18:1) IS	788.617	[M+H]+	**10.19**
[GP] Phosphatidylcholines	PC (36:2)	D31-PC(16:0/18:1) IS	786.601	[M+H]+	**9.32**
[GP] Phosphatidylcholines	PC (36:3)	D31-PC(16:0/18:1) IS	784.586	[M+H]+	**8.35**
[GP] Phosphatidylcholines	PC (36:4)	D31-PC(16:0/18:1) IS	782.570	[M+H]+	**8.01**
[GP] Phosphatidylcholines	PC (36:5)	D31-PC(16:0/18:1) IS	780.554	[M+H]+	**7.13**

[GP] Phosphatidylcholines	PC (38:0)	D31-PC(16:0/18:1) IS	818.664	[M+H]+	**12.13**
[GP] Phosphatidylcholines	PC (38:1)	D31-PC(16:0/18:1) IS	816.648	[M+H]+	**11.22**
[GP] Phosphatidylcholines	PC (38:2)	D31-PC(16:0/18:1) IS	814.633	[M+H]+	**10.44**
[GP] Phosphatidylcholines	PC (38:3)	D31-PC(16:0/18:1) IS	812.617	[M+H]+	**9.82**
[GP] Phosphatidylcholines	PC (38:4)	D31-PC(16:0/18:1) IS	810.601	[M+H]+	**9.41**
[GP] Phosphatidylcholines	PC (40:0)	D31-PC(16:0/18:1) IS	846.695	[M+H]+	**13.20**
[GP] Phosphatidylcholines	PC (40:1)	D31-PC(16:0/18:1) IS	844.680	[M+H]+	**12.29**
[GP] Phosphatidylcholines	PC (40:2)	D31-PC(16:0/18:1) IS	842.664	[M+H]+	**11.60**
[GP] Phosphatidylcholines	PC (40:3)	D31-PC(16:0/18:1) IS	840.648	[M+H]+	**10.82**
[GP] Phosphatidylcholines	PC (40:4)	D31-PC(16:0/18:1) IS	838.632	[M+H]+	**10.29**
[GP] Phosphatidylcholines	PC (40:5)	D31-PC(16:0/18:1) IS	836.617	[M+H]+	**9.50**
[GP] Phosphatidylcholines	PC (42:2)	D31-PC(16:0/18:1) IS	870.695	[M+H]+	**12.63**
[GP] Phosphatidylcholines	PC (42:3)	D31-PC(16:0/18:1) IS	868.679	[M+H]+	**11.94**
[GP] Phosphatidylcholines	PC (42:4)	D31-PC(16:0/18:1) IS	866.664	[M+H]+	**11.19**
[GP] Lyso-phosphatidylcholine	**LPC**				
[GP] Lyso-phosphatidylcholine	LPC (16:0)	LPC (17:0) IS	496.340	[M+H]+	**2.44**
[GP] Lyso-phosphatidylcholine	LPC (16:1)	LPC (17:0) IS	494.325	[M+H]+	**2.10**
[GP] Lyso-phosphatidylcholine	LPC (18:0)	LPC (17:0) IS	524.372	[M+H]+	**3.19**
[GP] Lyso-phosphatidylcholine	LPC (18:1)	LPC (17:0) IS	522.356	[M+H]+	**2.73**
[GP] Lyso-phosphatidylcholine	LPC (18:2)	LPC (17:0) IS	520.340	[M+H]+	**2.29**
[GP] Lyso-phosphatidylcholine	LPC (20:1)	LPC (17:0) IS	550.387	[M+H]+	**3.35**
[GP] Lyso-phosphatidylcholine	LPC (20:2)	LPC (17:0) IS	548.372	[M+H]+	**2.88**
[GP] Phospatidylethanolamines	**PE**				
[GP] Phospatidylethanolamines	PE (32:0)	D31-PE (16:0/18:1) IS	692.523	[M+H]+	**8.41**
[GP] Phospatidylethanolamines	PE (32:1)	D31-PE (16:0/18:1) IS	690.507	[M+H]+	**7.44**
[GP] Phospatidylethanolamines	PE (34:0)	D31-PE (16:0/18:1) IS	720.554	[M+H]+	**9.79**
[GP] Phospatidylethanolamines	PE (34:1)	D31-PE (16:0/18:1) IS	718.539	[M+H]+	**8.85**
[GP] Phospatidylethanolamines	PE (34:2)	D31-PE (16:0/18:1) IS	716.523	[M+H]+	**7.91**
[GP] Phospatidylethanolamines	PE (36:1)	D31-PE (16:0/18:1) IS	746.570	[M+H]+	**10.19**
[GP] Phospatidylethanolamines	PE (36:2)	D31-PE (16:0/18:1) IS	744.554	[M+H]+	**9.32**
[GP] Phospatidylethanolamines	PE (36:4)	D31-PE (16:0/18:1) IS	740.523	[M+H]+	**8.01**

[GP] Lyso-phosphatidylethanolamines	**LPE**				
[GP] Lyso-phosphatidylethanolamines	LPE (18:0)	LPE (17:1) IS	482.325	[M+H]+	**3.19**
[GP] Lyso-phosphatidylethanolamines	LPE (18:1)	LPE (17:1) IS	480.309	[M+H]+	**2.73**
[GP] Lyso-phosphatidylethanolamines	LPE (18:2)	LPE (17:1) IS	478.293	[M+H]+	**2.38**
[GP] Phosphatidylserines	**PS**				
[GP] Phosphatidylserines	PS (34:1)	D31-PS (16:0/18:1) IS	762.529	[M+H]+	**8.22**
[GP] Phosphatidylserines	PS (34:2)	D31-PS (16:0/18:1) IS	760.511	[M+H]+	**7.26**
[GP] Phosphatidylserines	PS (36:1)	D31-PS (16:0/18:1) IS	790.560	[M+H]+	**9.60**
[GP] Phosphatidylserines	PS (36:2)	D31-PS (16:0/18:1) IS	788.543	[M+H]+	**8.69**
[GP] Phosphatidylserines	PS (36:3)	D31-PS (16:0/18:1) IS	786.529	[M+H]+	**7.76**
[GP] Phosphatidylserines	PS (38:1)	D31-PS (16:0/18:1) IS	818.591	[M+H]+	**10.85**
[GP] Phosphatidylserines	PS (38:2)	D31-PS (16:0/18:1) IS	816.575	[M+H]+	**9.88**
[GP] Phosphatidylserines	PS (40:1)	D31-PS (16:0/18:1) IS	846.622	[M+H]+	**11.98**
[GP] Phosphatidylserines	PS (40:2)	D31-PS (16:0/18:1) IS	844.607	[M+H]+	**11.01**
[GP] Phosphatidylserines	PS (40:4)	D31-PS (16:0/18:1) IS	840.575	[M+H]+	**9.66**
[GP] Phosphatidylserines	PS (40:5)	D31-PS (16:0/18:1) IS	838.560	[M+H]+	**8.97**
[GP] Phosphatidylserines	PS (40:6)	D31-PS (16:0/18:1) IS	836.544	[M+H]+	**8.57**
[GP] Lyso-phosphatidylserines	**LPS**				
[GP] Lyso-phosphatidylserines	LPS (18:0)	LPS (17:1) IS	526.314	[M+H]+	**3.01**
[GP] Phosphatidylglycerol	**PG**				
[GP] Phosphatidylglycerol	PG (34:1)	D31-PG (16:0/18:1) IS	766.560	[M+NH4]+	**7.44**
[GP] Phosphatidylglycerol	PG (34:2)	D31-PG (16:0/18:1) IS	764.544	[M+NH4]+	**6.63**
[GL] Triacylglycerols	**TAG**				
[GL] Triacylglycerols	TAG (42:0)	TAG (17:0/17:0/17:0) IS	740.677	[M+NH4]+	**14.60**
[GL] Triacylglycerols	TAG (42:1)	TAG (17:0/17:0/17:0) IS	738.661	[M+NH4]+	**14.01**
[GL] Triacylglycerols	TAG (42:2)	TAG (17:0/17:0/17:0) IS	736.646	[M+NH4]+	**13.38**
[GL] Triacylglycerols	TAG (44:0)	TAG (17:0/17:0/17:0) IS	768.708	[M+NH4]+	**15.38**
[GL] Triacylglycerols	TAG (44:1)	TAG (17:0/17:0/17:0) IS	766.693	[M+NH4]+	**14.82**
[GL] Triacylglycerols	TAG (44:2)	TAG (17:0/17:0/17:0) IS	764.677	[M+NH4]+	**14.26**
[GL] Triacylglycerols	TAG (46:0)	TAG (17:0/17:0/17:0) IS	796.739	[M+NH4]+	**16.04**
[GL] Triacylglycerols	TAG (46:1)	TAG (17:0/17:0/17:0) IS	794.724	[M+NH4]+	**15.57**
[GL] Triacylglycerols	TAG (46:2)	TAG	792.708	[M+NH4]	**15.07**

		(17:0/17:0/17:0) IS		+	
[GL] Triacylglycerols	TAG (46:3)	TAG (17:0/17:0/17:0) IS	790.693	[M+NH4]+	**14.51**
[GL] Triacylglycerols	TAG (48:0)	TAG (17:0/17:0/17:0) IS	824.770	[M+NH4]+	**16.76**
[GL] Triacylglycerols	TAG (48:1)	TAG (17:0/17:0/17:0) IS	822.757	[M+NH4]+	**16.23**
[GL] Triacylglycerols	TAG (48:2)	TAG (17:0/17:0/17:0) IS	820.739	[M+NH4]+	**15.76**
[GL] Triacylglycerols	TAG (48:3)	TAG (17:0/17:0/17:0) IS	818.724	[M+NH4]+	**15.26**
[GL] Triacylglycerols	TAG (48:4)	TAG (17:0/17:0/17:0) IS	816.708	[M+NH4]+	**14.73**
[GL] Triacylglycerols	TAG (49:1)	TAG (17:0/17:0/17:0) IS	836.740	[M+NH4]+	**16.54**
[GL] Triacylglycerols	TAG (49:2)	TAG (17:0/17:0/17:0) IS	834.725	[M+NH4]+	**16.07**
[GL] Triacylglycerols	TAG (50:0)	TAG (17:0/17:0/17:0) IS	852.772	[M+NH4]+	**17.48**
[GL] Triacylglycerols	TAG (50:1)	TAG (17:0/17:0/17:0) IS	850.756	[M+NH4]+	**16.95**
[GL] Triacylglycerols	TAG (50:2)	TAG (17:0/17:0/17:0) IS	848.740	[M+NH4]+	**16.45**
[GL] Triacylglycerols	TAG (50:3)	TAG (17:0/17:0/17:0) IS	846.725	[M+NH4]+	**15.98**
[GL] Triacylglycerols	TAG (50:4)	TAG (17:0/17:0/17:0) IS	844.709	[M+NH4]+	**15.41**
[GL] Triacylglycerols	TAG (50:5)	TAG (17:0/17:0/17:0) IS	842.693	[M+NH4]+	**14.91**
[GL] Triacylglycerols	TAG (51:1)	TAG (17:0/17:0/17:0) IS	864.772	[M+NH4]+	**17.26**
[GL] Triacylglycerols	TAG (51:2)	TAG (17:0/17:0/17:0) IS	862.756	[M+NH4]+	**16.76**
[GL] Triacylglycerols	TAG (51:3)	TAG (17:0/17:0/17:0) IS	860.740	[M+NH4]+	**16.29**
[GL] Triacylglycerols	TAG (51:4)	TAG (17:0/17:0/17:0) IS	858.725	[M+NH4]+	**15.82**
[GL] Triacylglycerols	TAG (52:0)	TAG (17:0/17:0/17:0) IS	880.803	[M+NH4]+	**18.32**
[GL] Triacylglycerols	TAG (52:1)	TAG (17:0/17:0/17:0) IS	878.787	[M+NH4]+	**17.70**
[GL] Triacylglycerols	TAG (52:2)	TAG (17:0/17:0/17:0) IS	876.772	[M+NH4]+	**17.13**
[GL] Triacylglycerols	TAG (52:3)	TAG (17:0/17:0/17:0) IS	874.756	[M+NH4]+	**16.63**
[GL] Triacylglycerols	TAG (52:4)	TAG (17:0/17:0/17:0) IS	872.740	[M+NH4]+	**16.17**
[GL] Triacylglycerols	TAG (52:5)	TAG (17:0/17:0/17:0) IS	870.725	[M+NH4]+	**15.67**
[GL] Triacylglycerols	TAG (53:2)	TAG (17:0/17:0/17:0) IS	890.787	[M+NH4]+	**17.48**
[GL] Triacylglycerols	TAG (53:3)	TAG (17:0/17:0/17:0) IS	888.772	[M+NH4]+	**16.98**
[GL] Triacylglycerols	TAG (53:4)	TAG (17:0/17:0/17:0) IS	886.756	[M+NH4]+	**16.48**
[GL] Triacylglycerols	TAG (53:5)	TAG (17:0/17:0/17:0) IS	884.740	[M+NH4]+	**16.01**
[GL] Triacylglycerols	TAG (54:0)	TAG (17:0/17:0/17:0) IS	908.834	[M+NH4]+	**19.29**
[GL] Triacylglycerols	TAG (54:1)	TAG (17:0/17:0/17:0) IS	906.818	[M+NH4]+	**18.51**
[GL] Triacylglycerols	TAG (54:2)	TAG	904.803	[M+NH4]	**17.92**

		(17:0/17:0/17:0) IS		+	
[GL] Triacylglycerols	TAG (54:3)	TAG (17:0/17:0/17:0) IS	902.787	[M+NH4] +	**17.32**
[GL] Triacylglycerols	TAG (54:4)	TAG (17:0/17:0/17:0) IS	900.772	[M+NH4] +	**16.79**
[GL] Triacylglycerols	TAG (54:5)	TAG (17:0/17:0/17:0) IS	898.756	[M+NH4] +	**16.32**
[GL] Triacylglycerols	TAG (54:6)	TAG (17:0/17:0/17:0) IS	896.740	[M+NH4] +	**15.82**
[GL] Triacylglycerols	TAG (54:7)	TAG (17:0/17:0/17:0) IS	894.725	[M+NH4] +	**15.70**
[GL] Triacylglycerols	TAG (56:2)	TAG (17:0/17:0/17:0) IS	932.834	[M+NH4] +	**18.73**
[GL] Triacylglycerols	TAG (56:3)	TAG (17:0/17:0/17:0) IS	930.819	[M+NH4] +	**18.07**
[GL] Triacylglycerols	TAG (56:4)	TAG (17:0/17:0/17:0) IS	928.833	[M+NH4] +	**17.48**
[GL] Triacylglycerols	TAG (56:5)	TAG (17:0/17:0/17:0) IS	926.818	[M+NH4] +	**17.01**
[GL] Triacylglycerols	TAG (56:7)	TAG (17:0/17:0/17:0) IS	922.786	[M+NH4] +	**16.35**
[GL] Triacylglycerols	TAG (56:8)	TAG (17:0/17:0/17:0) IS	920.771	[M+NH4] +	**15.85**
[GL] Triacylglycerols	TAG (58:8)	TAG (17:0/17:0/17:0) IS	948.802	[M+NH4] +	**16.54**
[ST] Cholesteryl ester	**CE**				
[ST] Cholesteryl ester	CE (16:0)	CE (17:0) IS	642.589	[M+NH4] +	**16.79**
[ST] Cholesteryl ester	CE (16:1)	CE (17:0) IS	640.573	[M+NH4] +	**16.17**
[ST] Cholesteryl ester	CE (18:0)	CE (17:0) IS	670.620	[M+NH4] +	**17.76**
[ST] Cholesteryl ester	CE (18:1)	CE (17:0) IS	668.604	[M+NH4] +	**16.98**
[ST] Cholesteryl ester	CE (18:2)	CE (17:0) IS	666.589	[M+NH4] +	**16.35**
[ST] Cholesteryl ester	CE (18:3)	CE (17:0) IS	664.576	[M+NH4] +	**15.76**
[ST] Cholesteryl ester	CE (20:3)	CE (17:0) IS	692.605	[M+NH4] +	**16.54**
[ST] Cholesteryl ester	CE (20:4)	CE (17:0) IS	690.589	[M+NH4] +	**16.04**
[ST] Cholesteryl ester	CE (20:5)	CE (17:0) IS	688.574	[M+NH4] +	**15.51**

Table S2. FTIR band position and assignment in human, pig and mice right ventricle samples.

Band position (cm^{-1})			Assignment
**human**	**pig**	**mice**	
3280	3280	3280	Amide A<br>Mainly the ν(N-H) mode of proteins with the contribution of the ν(O-H) stretching mode of H_2O and polysaccharides
3072	3072	3072	ν(C-H) aromatic
3011	3011	3011	ν(C=H) of unsaturated lipids, triglycerides, fatty acids
2955, 2922, 2871, 2852	2957, 2923, 2871, 2852	2958, 2923, 2871, 2853	$\nu_{as}(CH_3)$, $\nu_{as}(CH_2)$,<br>$\nu_s(CH_3)$,$\nu_s(CH_2)$,<br>(Gly, Pro, Hyp, and Ala) of proteins + phospholipids, triglycerides<br>Most representative of proteins: $\nu_s(CH_3)$<br>Most representative of lipids: $\nu_{as}(CH_2)$ and $\nu_s(CH_2)$
1743	1740 (weak)	1740 (weak)	ν(C=O) of the **ester carbonyl groups** of **phospholipids and triglycerides**
1712 (weak)	1712 (weak)	1712 (weak)	ν(C=O) of the nucleoside side of nucleic acids and **free fatty acids**
1644	1646	1646	Amide I ν(C=O)<br>Multi-component band sensitive to protein secondary structure
1538	1540	1538	Amide II ν(C-N), δ(N-H)<br>Multi-component band sensitive to protein secondary structure
1515	1515	1517	Tyrosine band
1463, 1456	1471, 1452	1463, 1454	$\delta(CH_2)$ scissoring, $\delta(CH_3)$ bending of lipids and proteins
1392	1392	1392	νs(COO^-) of free amino acids, fatty acids, $\delta(CH_3)$
1338	1338	1338	$\delta(CH_2)$ wagging of the proline chain (mainly collagen)
1315-1310<br>1302<br>1236	1315-1310<br>1302<br>1234	1315-1310<br>1302<br>1232	ECM specific<br>Myofibers specific<br>Overlapping bands of<br>amide III δplan(N-H) and ν(C-N) of proteins<br>1246-1235 cm^{-1}: $\nu_{as}(PO_2^-)$ stretching of phospholipids, nucleic acids<br>**1226 cm^{-1} : $\nu(SO_4^{2-})$ of proteoglycans**
1200-1000<br><br>1171-1154<br>1159<br><br>1120<br>1100-1090<br>1080	1200-1000<br><br>1171<br>1159<br>1120<br>1100-1090<br>1079	1200-1000<br><br>1171<br><br>1120 (weak)<br><br>1078	Overlapping bands of ν(C-O), ν(C-C), ν(C-OH), ν(C-O-C) of proteins, oligosaccharides, glycolipids, and $\nu_s(PO_2^-)$ of nucleic acids, phospholipids<br><br>ν_{as} (CO-O-C) of cholesterol esters, phospholipids<br>ν(C-OH) of Hydroxyproline residue (mainly) and nucleic acids<br>ν(C-O) lactate, polysaccharides (glycogen)<br>$\nu_s(PO_2^-)$ phospholipids<br>**ν(C-O-C) of collagen, glycogen, oligosaccharides, glycolipids, and proteoglycans (specific to the ECM)**

1043	1045	1044	νs(CO-O-C) of carbohydrates residues and polysaccharides
972	972	973	DNA, RNA, ribose–phosphate skeletal motions
929	929	929	ν(Cα-C)characteristic of α helices, Z-DNA

Table S3. FTIR band position and assignment in right and left ventricles and in left infarcted ventricle from human explanted ischemic hearts

**Band position (cm^{-1})**			**Assignment**
**RV**	**LV**	**LV INF**	
3280	3280	3280	Amide A, mainly the ν(N-H) mode of proteins with the contribution of the ν(O-H) stretching mode of H_2O and polysaccharides
3072	3072	3072	ν(C-H) aromatic
3011	3011	3006	ν(C=H) of unsaturated lipids, triglycerides, fatty acids
2955, 2922, 2871, 2852	2955,2922,2871,2852	2953,2921,2871, 2852 intense	$\nu_{as}(CH_3)$, $\nu_{as}(CH_2)$, $\nu_s(CH_3)$,$\nu_s(CH_2)$, (Gly, Pro, Hyp, and Ala) of proteins + phospholipids, triglycerides<br>Most representative of proteins: $\nu_s(CH_3)$<br>Most representative of lipids: $\nu_{as}(CH_2)$ and $\nu_s(CH_2)$
1743	1743	1743 intense	ν(C=O) of the ester carbonyl groups of phospholipids and triglycerides
1712	1712	1712	ν(C=O) of the nucleoside side of nucleic acids and free fatty acids
1644	1644	1644	Amide I ν(C=O)
1538	1538	1538	Amide II ν(C-N), δ(N-H)
1515	1515	1515	Tyrosine band
1463, 1456	1463, 1456	1463, 1456 more marked	$\delta(CH_2)$ scissoring, $\delta(CH_3)$ bending of lipids and proteins
1392	1392	1392 less marked	$\nu s(COO^-)$ of free amino acids, fatty acids, $\delta(CH_3)$
		1377 new band	
1338	1338	1338 More marked	$\delta(CH_2)$ wagging of the proline chain (mainly collagen)
1315-1310<br>1302<br><br>1236	1315-1310<br>1302<br><br>1236	1315-1310<br>1302<br>less marked<br>1237	ECM specific<br>Myofibers specific<br><br>Overlapping bands of amide III δplan(N-H) and ν(C-N) of proteins<br>1246-1235 cm^{-1}: $\nu as(PO_2^-)$ stretching of phospholipids, nucleic acids<br>1226 cm^{-1} : $\nu(SO_4^{2-})$ of proteoglycans

1200-1000	1200-1000	1200-1000	Overlapping bands of ν(C-O), ν(C-C), ν(C-OH), ν(C-O-C) of proteins, oligosaccharides, glycolipids, and $\nu_s(PO_2^-)$ of nucleic acids, phospholipids
1171	1171	1171<br>few marked	ν_{as}(CO-O-C) of cholesterol esters, phospholipids
1159	1159	1159 (intense)	
		1140 new band	ν(C-OH) of Hydroproline residue (mainly) and nucleic acids
1120	1120	1120<br>more marked	undefined
		1095 new band	ν(C-O) lactate, polysaccharides (glycogen)
1080	1080	1082	undefined
1044	1044	1038	ν(C-O-C) of collagen, glycogen, oligosaccharides, glycolipids, and proteoglycans (specific to the ECM)<br>νs(CO-O-C) of carbohydrates residues and polysaccharides
972	972	972<br>less marked	Phosphorylated proteins, phospholipids, and nucleic acids
929	929	929<br>less marked	ν(Cα-C)characteristic of α helices and Z-DNA

RV: right ventricle, LV: left ventricle, LV INF: infarcted left ventricle

Table S4. Levels of sphingolipids species (pmol equiv/mg prot) in right,left ventricles and left infarcted ventricle from human explanted ischemic hearts.

Variable	RV	LV	LV INF	*P vs* LV	*P vs* RV
SM	8757.8 ± 1355.5	8407.3 ± 796	8678.5 ± 1077.6	0.923	1
SM(d18:1/16:0)	1225.6 ± 143.1	1209.6 ± 118.8	1215.5 ± 218.6	1	1
SM(d18:1/18:1)	445.3 ± 166.4	404 ± 159.7	409.5 ± 96	1	0.483
SM(d18:1/20:0)	915.1 ± 199.2	820 ± 187	813.7 ± 153.5	1	0.216
SM(d18:1/20:1)	217.1 ± 39.1	200.4 ± 80.5	191.3 ± 39.2	1	0.09
SM(d18:1/22:0)	953.4 ± 201.9	910.5 ± 88.2	889.9 ± 185.7	1	0.472
SM(d18:1/22:1)	633.3 ± 182.6	563.6 ± 170.4	641.9 ± 161.8	0.556	1
SM(d18:1/24:0)	795.7 ± 158.5	775.1 ± 84.8	822.4 ± 114.8	0.405	0.95
SM(d18:1/24:1)	1030.4 ± 116.9	1089 ± 301.5	1005.1 ± 144.4	0.585	0.89
SM(d18:1/24:2)	817.4 ± 170.1	780 ± 156.3	847.5 ± 135.4	0.704	0.93
SM(d18:1/24:3)	49.7 ± 26	44.1 ± 15.5	62.4 ± 24	0.15	0.067
Cer(d18:1/14:0)	1.6 ± 0.3	1.5 ± 0.6	1.6 ± 0.6	0.951	1
Cer(d18:1/14:1)	2.2 ± 0.2	3.6 ± 4	2.1 ± 0.3	0.457	0.606
Cer(d18:1/16:0)	35.6 ± 11.3	26.8 ± 18.9	37.2 ± 24.7	0.183	1
Cer(d18:1/16:1)	2.5 ± 0.7	2 ± 0.8	2.4 ± 0.9	0.224	1
Cer(d18:1/22:0)	100.6 ± 48.5	79.5 ± 48.8	68.5 ± 42.9	0.644	0.051
Cer(d18:1/24:0)	189.2 ± 68.5	161.7 ± 58.9	156.6 ± 56	1	0.134
Cer(d18:1/24:1)	259.3 ± 121.8	200.6 ± 93.7	180 ± 53.4	0.878	0.081
dhSM	325.9 ± 61.6	364.7 ± 123.5	392.3 ± 74.6	0.833	0.063
dhSM(d18:0/14:0)	3.2 ± 1	3.2 ± 0.9	4.1 ± 1.4	0.226	0.094
dhSM(d18:0/20:0)	9 ± 1.8	12.1 ± 7.2	13 ± 6.9	1	0.217
dhSM(d18:0/22:0)	45.1 ± 12.1	40.9 ± 14.4	45.2 ± 11.3	0.642	1
dhSM(d18:0/24:0)	43 ± 18.6	51.5 ± 27.7	39.2 ± 16.8	0.206	0.836
CDH	39.4 ± 16.5	36.9 ± 15	51.2 ± 21.4	0.213	0.185

CDH(d18:1/14:0)	2 ± 0.7	2.3 ± 0.6	2.5 ± 0.6	0.73	0.129
CDH(d18:1/16:0)	7.5 ± 2.3	7.4 ± 2.5	11 ± 5.9	0.171	0.11
CDH(d18:1/18:0)	1.6 ± 0.7	1.4 ± 0.3	1.5 ± 0.5	1	0.978
CDH(d18:1/20:0)	1 ± 0.7	0.8 ± 0.3	0.8 ± 0.3	0.239	0.682
CDH(d18:1/22:0)	5.9 ± 3.8	5.5 ± 2.8	6.1 ± 2.9	1	1
CDH(d18:1/24:0)	7.8 ± 3.5	8.6 ± 3.9	12.7 ± 6.4	0.122	0.072
CDH(d18:1/24:1)	13.6 ± 7.7	12.7 ± 4.9	16.5 ± 9	0.68	0.528

Data are expressed as mean ± SD, n=9 (RV, LV and LV INF). SM: sphingomyelin, Cer: Ceramide, dhSM: dihydrosphingomyelin, CDH: ceramide dihexoside, SD: standard deviation, RV: right ventricle, LV: left ventricle, LV INF: infarcted left ventricle.

Table S5. Levels of glycerophospholipid species (pmol equiv/mg prot) in right,left ventricles and left infarcted ventricle from human explanted ischemic hearts.

Variable	Mean RV ± SD	Mean LV ± SD	Mean LV INF ± SD	P *vs* LV	P *vs* RV
PC(32:0)	4532.7 ± 1035.1	4741.2 ± 868.5	3976.1 ± 603.5	0.068	0.338
PC(32:1)	1941 ± 679.8	1931 ± 486.5	1437.4 ± 488.7	0.066	0.214
PC(34:0)	852.1 ± 349.4	897.6 ± 298	822.5 ± 342.3	0.629	1
PC(36:0)	32 ± 9.8	33.7 ± 10.7	31.4 ± 15.4	0.976	1
PC(36:1)	2959.7 ± 1021.7	2963.7 ± 756.3	2869.4 ± 830.7	1	1
PC(36:4)	3907.2 ± 944.2	4196.5 ± 843.7	3302.1 ± 678.4	0.069	0.17
PC(38:0)	4.4 ± 2.7	4.7 ± 1.7	5 ± 2.7	0.997	0.959
PC(38:1)	25.6 ± 13.5	26.7 ± 11.4	25 ± 13	1	1
PC(38:2)	188.3 ± 99.7	196.9 ± 75.1	172.9 ± 84.3	0.446	0.908
PC(38:3)	272.1 ± 133.2	272.2 ± 121.9	324.1 ± 262.5	0.655	0.826
PC(38:4)	2278.6 ± 780.8	2393.6 ± 527.4	2175.2 ± 510.3	0.273	1
PC(40:0)	2.1 ± 1.1	2.2 ± 1.3	2.7 ± 1.7	0.345	0.255
PC(40:1)	5.9 ± 4.3	6.3 ± 5	5 ± 3.3	0.588	0.686
PC(40:2)	9.5 ± 8.7	10.2 ± 9.2	7.2 ± 5.5	0.491	0.565

PC(40:3)	16.2 ± 10	14.8 ± 8.4	13.6 ± 7.7	0.972	0.351
PC(40:4)	49 ± 27.1	60.2 ± 23.9	82 ± 60.2	0.385	0.173
PC(40:5)	178.6 ± 115.9	183.4 ± 90.1	151 ± 109.8	0.139	0.464
PC(42:2)	3.6 ± 2.9	3.5 ± 2.6	3.9 ± 3.1	1	1
PC(42:3)	5 ± 3.3	4.9 ± 2.7	4.8 ± 3.8	1	0.926
PC(42:4)	3.3 ± 2.1	4 ± 2.2	7.9 ± 7.4	0.4	0.919
LPC	5228.6 ± 1636.4	4563.5 ± 1085	4367.7 ± 1122	1	0.489
LPC(16:0)	1874 ± 666.9	1791.6 ± 372.5	1598.4 ± 378	0.358	0.606
LPC(16:1)	118.9 ± 31.1	107.9 ± 36.8	92.4 ± 43	0.602	0.413
LPC(18:0)	646 ± 339.1	575.6 ± 153.1	663.6 ± 202.2	0.274	1
LPC(18:1)	1371.1 ± 426.2	1316.6 ± 259.6	1179.4 ± 348.8	0.203	0.566
LPC(18:2)	1185.3 ± 681.1	742.5 ± 571.5	803 ± 572.7	1	0.152
LPC(20:1)	21.9 ± 9.6	17.1 ± 6.4	18.2 ± 7.8	0.871	0.481
LPC(20:2)	11.6 ± 4	12.2 ± 5.4	12.6 ± 4.3	1	0.758
PS	6458.8 ± 3646.8	6022.5 ± 2563.7	5060.2 ± 1901.3	0.553	0.584
PS(34:1)	37.5 ± 39.7	15.6 ± 19.8	32.2 ± 34.9	0.317	1
PS(34:2)	15.9 ± 15.7	8.3 ± 6.1	5.3 ± 4.4	0.149	0.521
PS(36:1)	2964.5 ± 1610	2716.5 ± 1106.5	2763.3 ± 1115.3	1	1
PS(36:2)	246.4 ± 167.8	220.2 ± 90.4	208.7 ± 88.1	1	0.898
PS(36:3)	11.6 ± 5.5	15.2 ± 7.1	11 ± 4.7	0.316	1
PS(38:1)	297.7 ± 221.1	256.2 ± 133.6	194.1 ± 97.4	0.433	0.448
PS(38:2)	227.6 ± 153.4	235.9 ± 114.4	159.7 ± 76.7	0.08	0.391
PS(40:1)	414.9 ± 325.7	411 ± 343.5	210.7 ± 103.4	0.2	0.185
PS(40:2)	404 ± 246.6	368.9 ± 222.7	225.9 ± 139.9	0.154	0.169
PS(40:4)	193 ± 110.2	210.1 ± 70.2	230.8 ± 155.3	1	0.832
PS(40:5)	91.7 ± 44.9	108.1 ± 29	88 ± 33.5	0.311	1
PS(40:6)	1567 ± 982	1461.2 ± 785.3	931.7 ± 507.5	0.208	0.194

LPS	852.4 ± 324.9	799.3 ± 237.2	817.8 ± 224	1	1
LPS(18:0)	852.4 ± 324.9	799.3 ± 237.2	817.8 ± 224	1	1
PE(32:0)	83.9 ± 29.4	89.7 ± 37.4	70.4 ± 28.4	0.144	0.625
PE(32:1)	62.6 ± 23.4	63.1 ± 26.5	51.5 ± 23.3	0.462	0.593
PE(34:0)	339 ± 222	378.9 ± 201.9	353.1 ± 332.9	1	1
PE(34:1)	258.7 ± 100.1	262.1 ± 110.8	261 ± 55.2	1	1
PE(36:1)	569.2 ± 271.1	613.5 ± 211.7	662.3 ± 359.5	0.945	0.442
PE(36:2)	971.5 ± 318.7	952.4 ± 263.7	820.4 ± 165.5	0.146	0.228
LPE	2061 ± 590.8	2204.5 ± 328.9	1880.3 ± 531.8	0.12	0.885
LPE(18:0)	1653.7 ± 500.6	1785 ± 245.8	1467.7 ± 485	0.069	0.777
LPE(18:1)	262.6 ± 115.3	276.8 ± 99.3	317.6 ± 88.9	0.587	0.361

Data are expressed as mean ± SD, n=9 (RV, LV and LV INF). PC: phosphatidylcholine, LPC : lysophosphatidylcholine, PS: phosphatidylserine, LPS: lysophosphatidylserine, PE: phosphatidylethanolamine, LPE : lysophosphatidylethanolamine. SD: standard deviation, RV: right ventricle, LV: left ventricle, LV INF: infarcted left ventricle.

Table S6. Levels of neutral lipid species (pmol equiv/mg prot) in right,left ventricles and left infarcted ventricle from human explanted ischemic hearts.

Variable	RV	LV	LV INF	P *vs* LV	P *vs* RV
TAG	1061930.8 ± 444128.8	845205.2 ± 306116	985256.6 ± 440806.9	0.607	0.998
TAG(42:0)	5868.2 ± 4901.8	3424.3 ± 2476	5356.2 ± 5509	0.579	1
TAG(42:1)	8004.3 ± 6399.6	6060.1 ± 8450.8	6504.1 ± 6898.1	1	1
TAG(42:2)	3697.7 ± 2965.4	2894 ± 4184.5	2997.6 ± 3515.3	1	1
TAG(44:0)	8540.2 ± 6701.6	4701.8 ± 2762.7	8290.7 ± 7035.6	0.203	1
TAG(44:1)	16852.4 ± 12806.9	10479.7 ± 9470.2	13784.7 ± 12025.9	0.859	0.896
TAG(44:2)	11871.9 ± 9765.8	8437 ± 10674.3	9062.8 ± 8817.9	1	0.825
TAG(46:0)	5330.9 ± 2672.5	3697.9 ± 1620.9	6058.5 ± 4341.6	0.139	0.776
TAG(46:1)	25525.8 ± 13733.1	17128.6 ± 8414.5	22505.7 ± 15052.5	0.465	0.868
TAG(46:2)	20283.1 ± 11364.1	16031.2 ± 11331.7	20428.5 ± 15090.9	0.729	1
TAG(46:3)	8966.3 ± 7953.1	5055.1 ± 5081.9	6565.9 ± 6705.2	0.959	0.725
TAG(48:0)	3396.7 ± 1331.2	3131.8 ± 1104	4062.1 ± 2614.8	0.319	0.499
TAG(48:1)	28448.1 ± 11185	21957 ± 6766.7	26419.6 ± 13188.7	0.5	1
TAG(48:2)	39580.8 ± 22594.5	26594.4 ± 12200.5	36621.9 ± 23208.2	0.352	1
TAG(48:3)	22601.9 ± 10829.7	15776.1 ± 10490.5	20080.3 ± 14974.4	0.699	1
TAG(48:4)	7316.2 ± 9348	3566.9 ± 3665.5	5405.4 ± 5715	0.632	0.749
TAG(49:1)	3262.5 ± 1686.8	2487.2 ± 880.9	3573.2 ± 1779	0.073	0.868
TAG(49:2)	3952.6 ± 2794.8	2807.8 ± 1518.5	4507.8 ± 2789.4	0.163	0.898
TAG(50:0)	2336.8 ± 1021	1975.4 ± 823.2	2531.9 ± 1751.2	0.672	1
TAG(50:1)	43657.6 ± 14628.5	36592.9 ± 7767.1	43135.8 ± 15947.9	0.404	1
TAG(50:2)	47495.7 ± 17739.2	37718.4 ± 7276.3	43679.4 ± 14951.2	0.473	0.871
TAG(50:3)	41952.9 ± 19280.9	30573 ± 13352	38315.1 ± 21025.3	0.562	0.997
TAG(50:4)	16693.6 ± 8800.6	12512.8 ± 9895.2	17956 ± 15858.8	0.581	1

TAG(50:5)	3247.2 ± 3867.6	1597.7 ± 1566.6	2256.6 ± 2335.1	0.766	0.602
TAG(51:1)	3927.4 ± 1538.6	3160.6 ± 1223.9	4075 ± 2081.4	0.194	1
TAG(51:2)	7647.1 ± 3739.1	6026 ± 2988.6	8626.2 ± 4817.8	0.191	0.795
TAG(51:3)	5623.7 ± 4037.2	4140.2 ± 2858.9	5729.8 ± 3979	0.428	1
TAG(51:4)	1645.8 ± 2186.4	1030.3 ± 807.3	1330.7 ± 1307.1	0.724	0.802
TAG(52:0)	713.8 ± 361.8	573.7 ± 251.4	1000.9 ± 1465.1	0.655	0.849
TAG(52:1)	43974.8 ± 16478.9	36653.7 ± 12914.8	41488.7 ± 16540.8	0.466	0.98
TAG(52:2)	71567.1 ± 22304.1	66550.3 ± 13683.2	70575.6 ± 22117.3	0.863	1
TAG(52:3)	62134.6 ± 23311.3	54207.9 ± 10499.3	57867.4 ± 14553.1	0.667	0.892
TAG(52:4)	46856.5 ± 24387	36234.6 ± 11865	40280.1 ± 17469	0.773	0.64
TAG(52:5)	19926 ± 16408.4	12679.3 ± 10363.9	16001 ± 12297.1	0.799	0.697
TAG(53:2)	15543.5 ± 9573.6	11109.3 ± 7023.1	15658.5 ± 10890	0.421	1
TAG(53:3)	10000.2 ± 6189.2	7249.4 ± 4044.3	8950.6 ± 5582.8	0.537	0.868
TAG(53:4)	2834.4 ± 1789.8	2392.1 ± 1737.2	2751.6 ± 1958.3	0.915	1
TAG(53:5)	667.8 ± 699.2	504.6 ± 335.1	672.5 ± 747.1	0.764	1
TAG(54:0)	134.9 ± 49.1	122.9 ± 41.4	271.4 ± 487.2	0.572	0.57
TAG(54:1)	24126.7 ± 12937.8	16749.9 ± 9902.9	22594.8 ± 14014.6	0.417	1
TAG(54:2)	69774.4 ± 23014.2	57635 ± 18860	65656.5 ± 27578	0.573	1
TAG(54:3)	84057.9 ± 25637.3	71895.2 ± 15075.4	77378.4 ± 22402.2	0.727	0.754
TAG(54:4)	59703.3 ± 19905.5	53874.5 ± 14102.1	64183.2 ± 27984.9	0.346	1
TAG(54:5)	36349.1 ± 16077.4	29631.4 ± 7161.4	31380.1 ± 11941	0.95	0.578
TAG(54:6)	11987.2 ± 7397.5	12366 ± 9736.4	11004.6 ± 7447.6	1	1
TAG(54:7)	4653.4 ± 4226.3	3356.2 ± 3810.6	2488.7 ± 1667.5	0.791	0.169
TAG(56:2)	19113.9 ± 12468.8	14030.6 ± 6803.7	16991.7 ± 12028.8	0.808	0.978
TAG(56:3)	31431.2 ± 15606.8	24525.8 ± 11174.5	26480.6 ± 15708.4	1	0.711
TAG(56:4)	19768.9 ± 9575.6	16572.2 ± 8686.2	18412.6 ± 11152	1	1
TAG(56:5)	10186.7 ± 6596.6	8381 ± 5246.8	8617.7 ± 5672.4	1	0.628

TAG(56:7)	9547.8 ± 5496.6	9082.8 ± 5787	7672.1 ± 3816.9	0.537	0.371
TAG(56:8)	6308.4 ± 5189.6	5904 ± 5882.6	5036.8 ± 3667.8	0.966	0.556
CHOL	20007.8 ± 6743.3	20016.8 ± 5508.9	23059.2 ± 6867.1	0.269	0.427
CE	8173.2 ± 5605.1	9512 ± 5421.2	12612.9 ± 10279.8	0.178	0.202
CE(16:0)	86.6 ± 82.2	119.4 ± 93.7	120.1 ± 115.7	1	0.727
CE(16:1)	121.9 ± 86.3	191.8 ± 121.9	283.7 ± 251.1	0.252	0.119
CE(18:0)	26.6 ± 15.4	63.3 ± 90.3	180.5 ± 270.3	0.207	0.23
CE(18:1)	1254.4 ± 1144.7	1860.6 ± 1278.1	1918.8 ± 2043.9	1	0.553
CE(18:2)	4776.8 ± 3110	5166.6 ± 2737.4	6001.3 ± 4998.4	0.561	0.35
CE(20:4)	1637.4 ± 1506.4	1758.1 ± 1327.3	3565.9 ± 3788	0.14	0.243

Data are expressed as mean ± SD, n=9 (RV, LV and LV INF). TAG: triacylglycerols, CHOL : cholesterol, CE: cholesteryl esters. SD : standard deviation, RV: right ventricle, LV: left ventricle, LV INF : infarced left ventricle.

Supplemental Figures

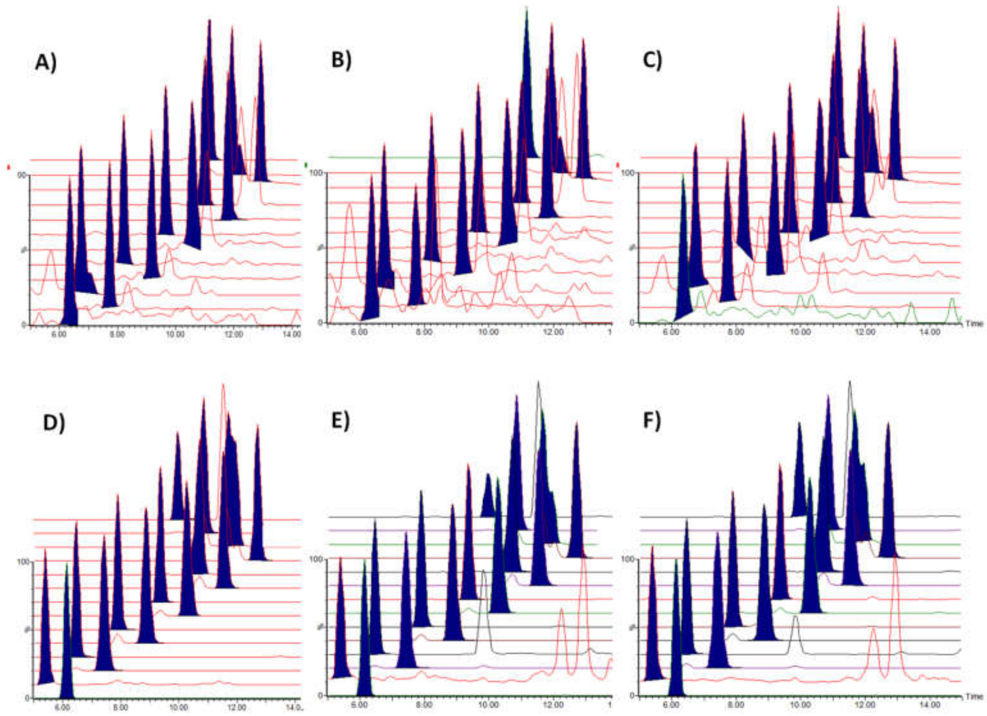

Figure S1. Representative ion chromatograms of ceramides (A-C) and sphingomyelins (D-F) detected in the right (A, D), left (B, E) and infarcted left ventricle (C, F) of the same patient. Trace values were indicated in Table S1.

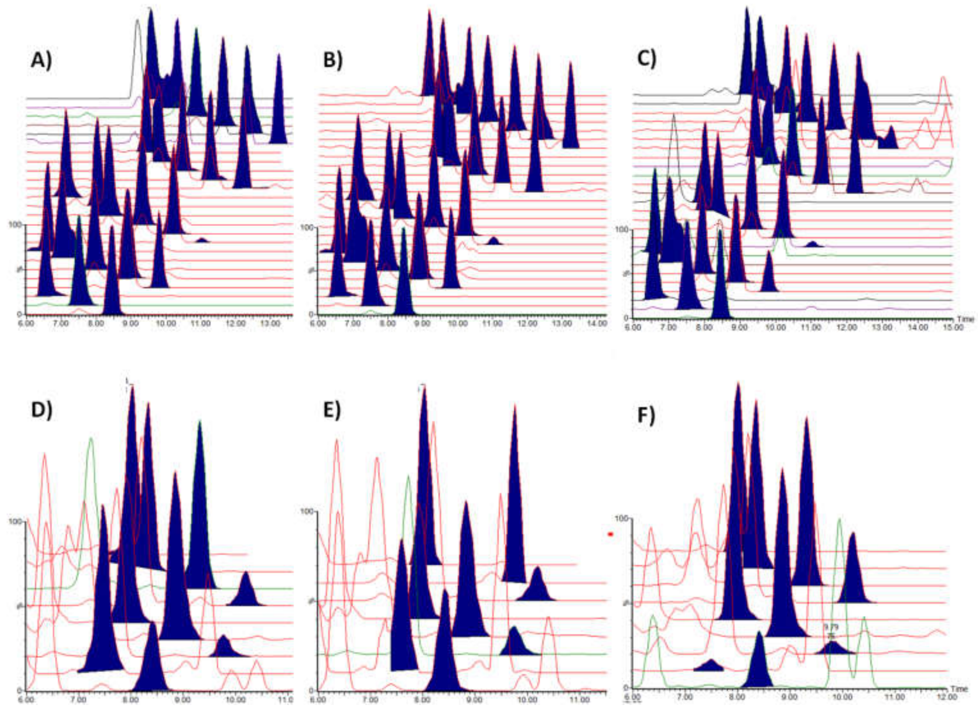

Figure S2. Representative ion chromatograms of phosphatidylcholines (PCs) (A-C) and phosphatidylethanolamines (Pes) (D-F) detected in the right (A, D), left (B, E) and infarcted left ventricle (C, F) of the same patient. Trace values were indicated in Table S1.

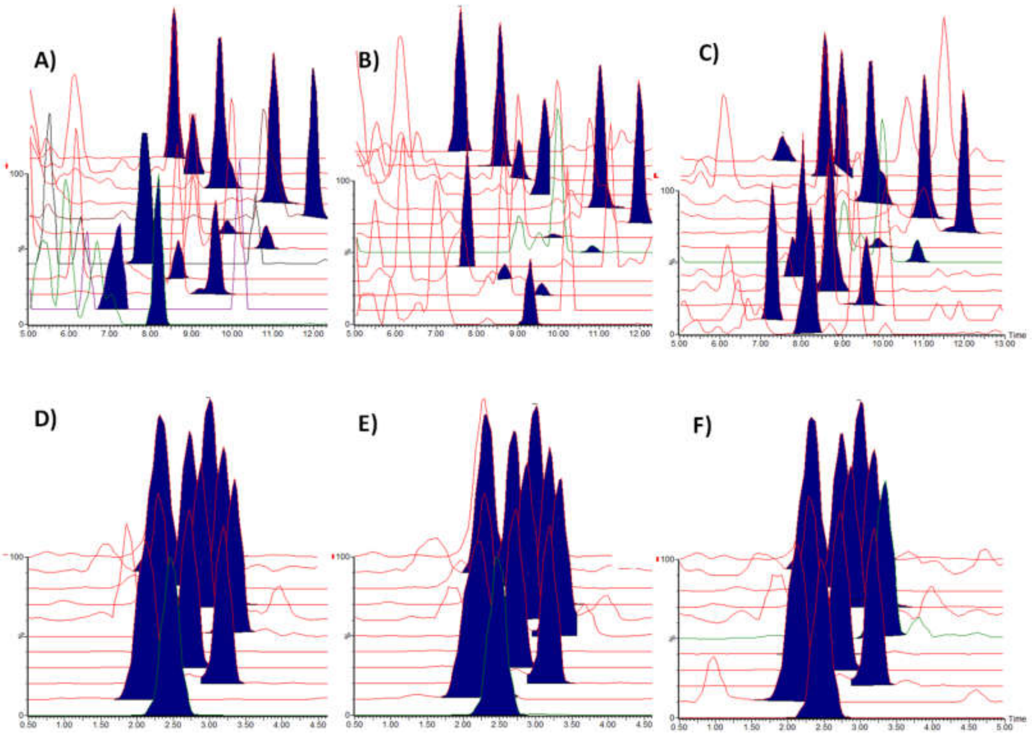

Figure S3. Representative ion chromatograms of phosphatidylserines (PSs) (A-C) and lyso-phospholipids (D-F) detected in the right (A, D), left (B, E) and infarcted left ventricle (C, F) of the same patient. D-F: From bottom to top lysophosphatidylcholines (lyso-PCs) (7 species), lysophosphatidlyethanolamines (lyso-PEs) (3 species) and lysophosphatidylserines (lyso-PS) (1 specie). Trace values were indicated in Table S1.

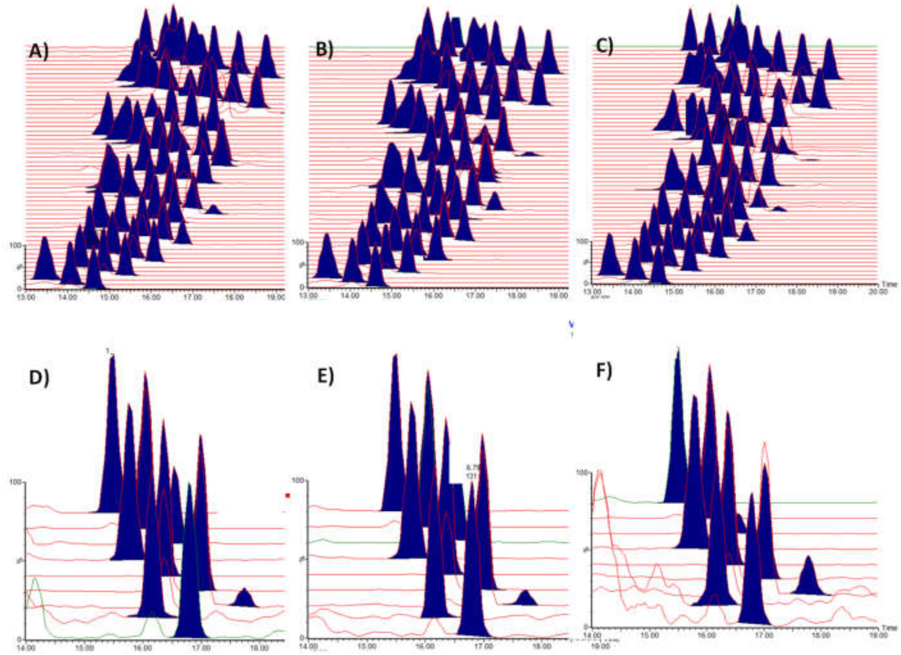

Figure S4. Representative ion chromatograms of triacylglycerols (TAGs) (A-C) and cholesteryl esters (CEs)(D-F) detected in the right (A, D), left (B, E) and infarcted left ventricle (C, F) of the same patient. Trace values were indicated in Table S1.

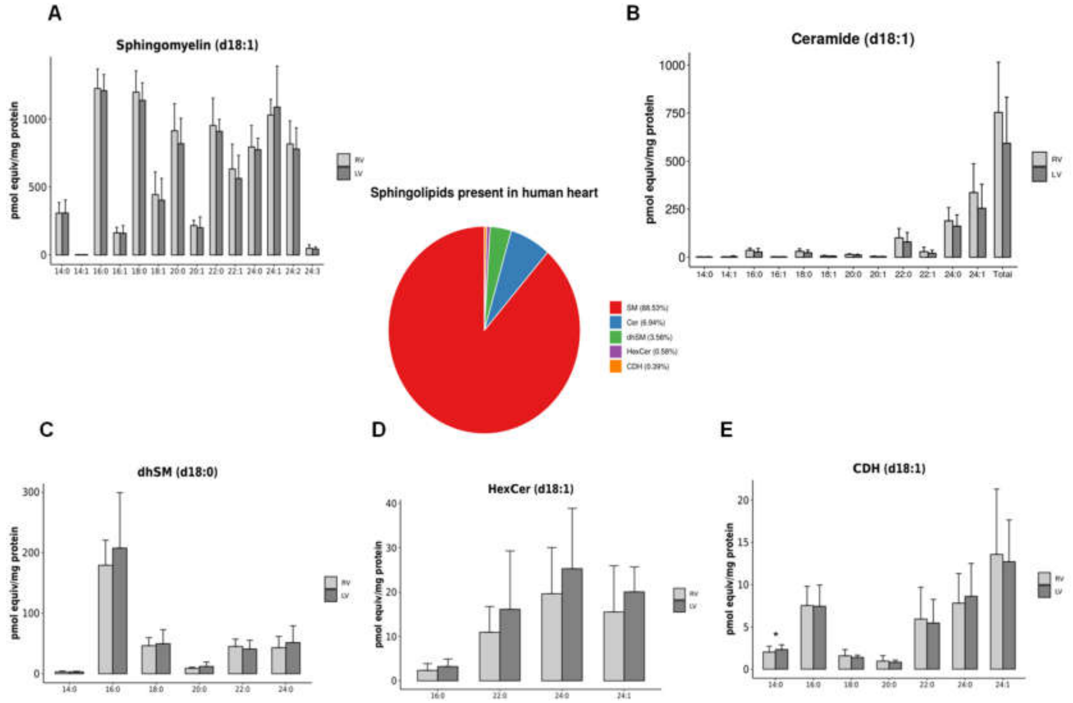

Figure S5. Levels of sphingolipids species in right and left ventricles from human explanted ischemic hearts. Bar graphs showing the levels of sphingomyelin (d18:1) (**A**), ceramide (d18:1) (**B**), dihydrosphingomyelin (dhSM d18:0) (**C**), hexosylceramide (HexCer d18:1) (**D**) and ceramide dihexoside (**E**). Data are expressed as mean ± SD , n = 9 . RV: right ventricle, LV: left ventricle. $p < 0.05$ *vs* RV

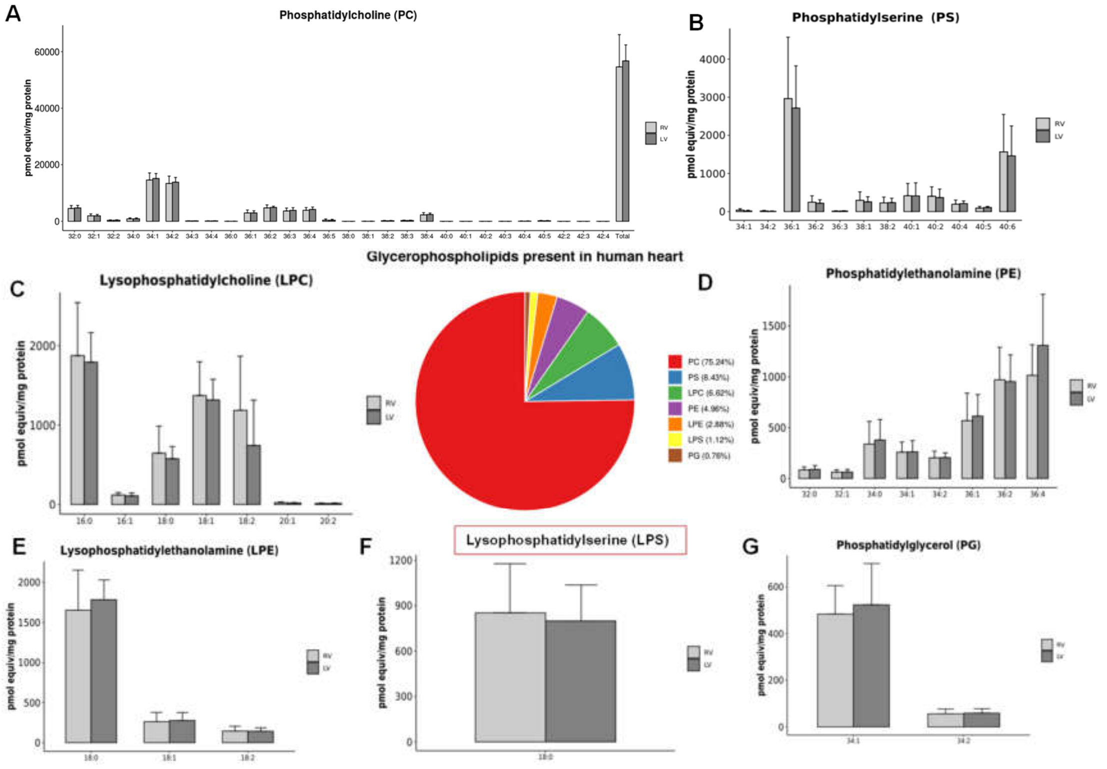

Figure S6. Levels of glycerophospholipid species in right and left ventricles from human explanted ischemic hearts. Bar graphs showing the levels of phosphatidylcholine (PC) (**A**), phosphatidylserine (PS) (**B**), lysophosphatidylcholine (LPC) (**C**), phosphatidylethanolamine (PE) (**D**), lysophosphatidylethanolamine (LPE), lysophosphatidylserine (LPS) (**F**), and phosphatidylglycerol (PG) (**G**). Data are expressed as mean ± SD , n = 9 . RV: right ventricle, LV: left ventricle.

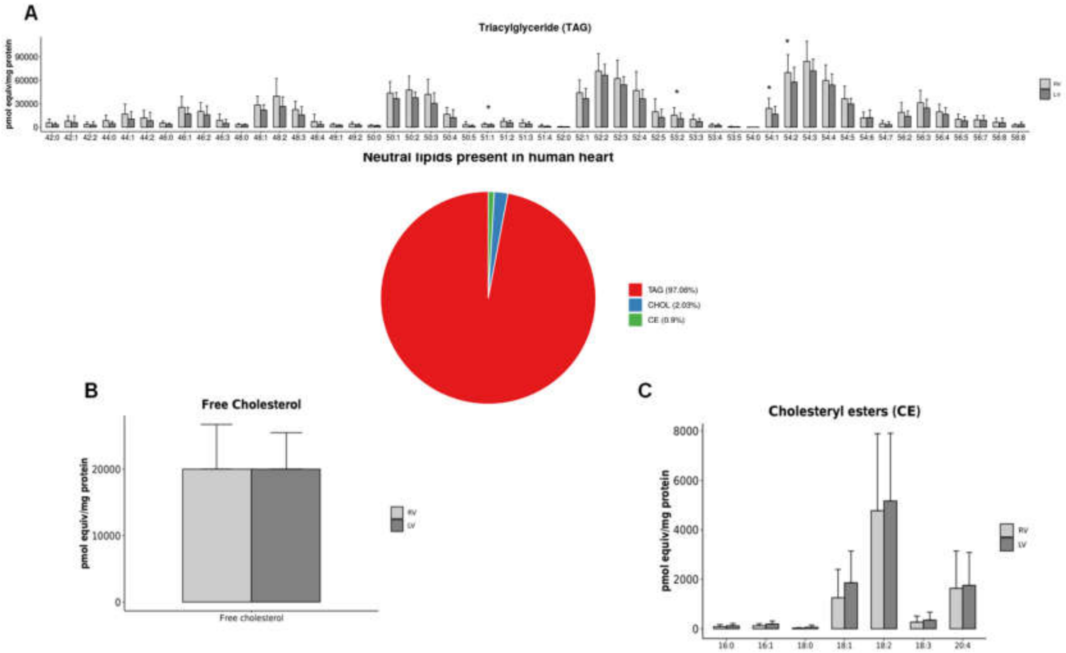

Figure S7. Levels neutral lipid species in right and left ventricles from human explanted ischemic hearts. Bar graphs showing the levels of triacylgycerols (TAG) (**A**), free cholesterol (**B**) and cholesteryl esters (CE) (**C**). Data are expressed as mean ± SD , n = 9 . RV: right ventricle, LV: left ventricle. *P<0.05 *vs* RV.

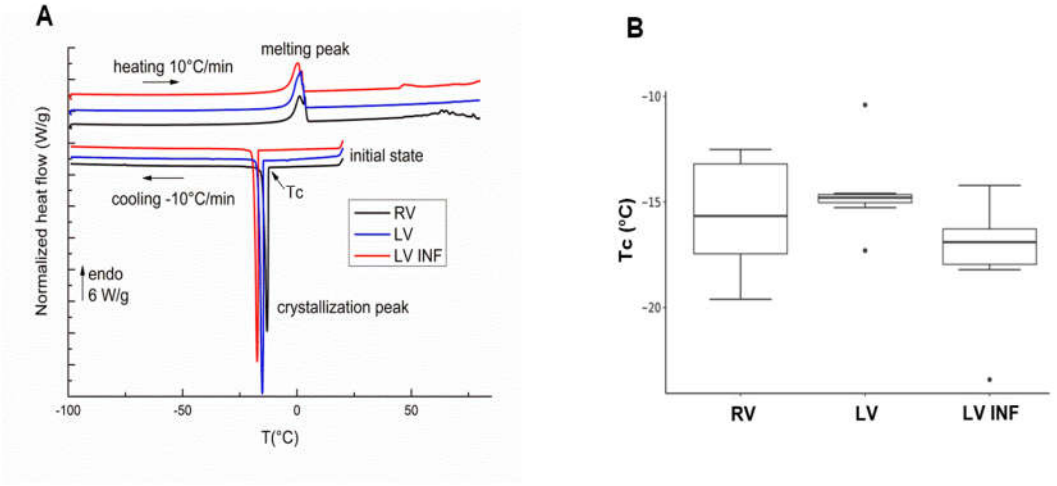

Figure S8. Hydric response of the right, left and left infarcted ventricles from human explanted ischemic hearts. Representative DSC thermograms (A). Tc: temperatura of water crystalization compiled from all cooling thermograms. Boxplots analysis of hydric response of right, left and left infacted ventricles in terms of temperature of water crystalization (Tc) (B). RV, right ventricle; LV, left ventricle; LV INF, infarcted left ventricle.
